# Analysis of proteome-wide degradation dynamics in ALS SOD1 iPSC-derived patient neurons reveals disrupted VCP homeostasis

**DOI:** 10.1016/j.celrep.2023.113160

**Published:** 2023-09-29

**Authors:** Konstantinos Tsioras, Kevin C. Smith, Seby L. Edassery, Mehraveh Garjani, Yichen Li, Chloe Williams, Elizabeth D. McKenna, Wenxuan Guo, Anika P. Wilen, Timothy J. Hark, Stefan L. Marklund, Lyle W. Ostrow, Jonathan D. Gilthorpe, Justin K. Ichida, Robert G. Kalb, Jeffrey N. Savas, Evangelos Kiskinis

**Affiliations:** 1 The Ken & Ruth Davee Department of Neurology, Northwestern University Feinberg School of Medicine, Chicago, IL 60611, USA; 2 Department of Stem Cell Biology and Regenerative Medicine, Eli and Edythe Broad Center for Regenerative Medicine and Stem Cell Research, Zilkha Neurogenetic Institute, University of Southern California, Keck School of Medicine, Los Angeles, CA 90033, USA; 3 Department of Integrative Medical Biology, Umeå University, 90187 Umeå, Sweden; 4 Department of Medical Biosciences, Clinical Chemistry, Umeå University, 90187 Umeå, Sweden; 5 Department of Neurology, Lewis Katz School of Medicine at Temple University, Philadelphia, PA, USA; 6 Simpson Querrey Institute, Northwestern University, Chicago, IL 60611, USA; 7 Department of Neuroscience, Northwestern University Feinberg School of Medicine, Chicago, IL 60611, USA; 8 Lead contact

## Abstract

Mutations in *SOD1* cause amyotrophic lateral sclerosis (ALS) through gain-of-function effects, yet the mechanisms by which misfolded mutant SOD1 (mutSOD1) protein impairs human motor neurons (MNs) remain unclear. Here, we use induced-pluripotent-stem-cell-derived MNs coupled to metabolic stable isotope labeling and mass spectrometry to investigate proteome-wide degradation dynamics. We find several proteins, including the ALS-causal valosin-containing protein (VCP), which predominantly acts in proteasome degradation and autophagy, that degrade slower in mutSOD1 relative to isogenic control MNs. The interactome of VCP is altered in mutSOD1 MNs *in vitro*, while VCP selectively accumulates in the affected motor cortex of ALS-SOD1 patients. Overexpression of VCP rescues mutSOD1 toxicity in MNs *in vitro* and in a *C*. *elegans* model *in vivo*, in part due to its ability to modulate the degradation of insoluble mutSOD1. Our results demonstrate that VCP contributes to mutSOD1-dependent degeneration, link two distinct ALS-causal genes, and highlight selective protein degradation impairment in ALS pathophysiology.

## In brief

Tsioras et al. use stable isotope labeling coupled with mass spectrometry to study proteome degradation dynamics in ALS SOD1 and isogenic control iPSC-derived neurons. VCP persists in mutSOD1 motor neurons; VCP can rescue mutant SOD1 toxicity *in vitro* and *in vivo* by modulating the degradation of insoluble SOD1.

## Graphical Abstract

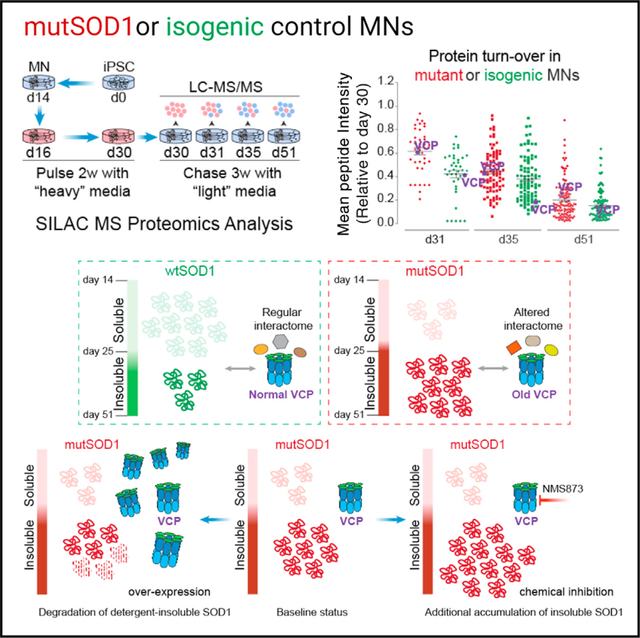

## INTRODUCTION

Amyotrophic lateral sclerosis (ALS) is a fatal neurodegenerative disease affecting upper and lower motor neurons (MNs) in the brain and spinal cord of the central nervous system. The damage in MNs leads to a progressive paralysis and eventually death due to respiratory failure.^1,2^ Most of ALS cases are sporadic, with approximately 10% exhibiting a familial pattern driven by strong monogenetic etiology.^3^ Up to 2% of all ALS cases result from mutations in the copper zinc superoxide dismutase 1 (*SOD1*) gene, which encodes a ubiquitously expressed enzyme responsible for the dismutation of superoxide radicals within the cytoplasm, nucleus, and intramembrane space of mitochondria.^4–7^ *SOD1* was the first gene reported to be genetically associated with ALS, originally identified through its autosomal dominant inheritance pattern.^8^

More than 200 *SOD1* mutations have been described in patients with ALS, and most of these are predicted to disrupt the conformational integrity of the holoprotein.^9,10^ Mutant SOD1 (mutSOD1) adopts a disordered conformation that is prone to misfolding, forming soluble oligomers and larger insoluble aggregates, a hallmark of SOD1-associated familial ALS.^11^ MutSOD1 aggregates prepared from both transgenic mice and human patients with ALS can cause prion-like propagation of toxic SOD1 aggregation and accelerate pathogenesis when inoculated into the spinal cord of transgenic SOD1 mice.^12–14^ Moreover, several studies based on mutSOD1 overexpression in animal and cell-culture models, as well as in induced pluripotent stem cell (iPSC)-derived neurons and human tissue post mortem, have highlighted pathological events that occur downstream of mutSOD1 including mitochondrial dysfunction, endoplasmic reticulum (ER) stress, and disruptions in neuronal excitability and cytoskeletal homeostasis.^15–22^ However, the precise mechanisms by which the accumulation of disordered or aggregated SOD1 protein impairs these pathways are not clear. Nevertheless, mutSOD1 is considered to drive disease pathogenesis, at least in part, via toxic gain-of-function effects. Centered around this premise, there are currently several therapeutic strategies in development with the goal of reducing the SOD1 expression level in patients.^23–26^ While antisense oligonucleotides targeting SOD1 mRNA demonstrate promising clinical efficacy,^27^ a better understanding of the impact of mutSOD1 protein on neuronal homeostasis is warranted.

Mutations in multiple other genes encoding proteins that are broadly involved in protein quality control pathways (e.g., *C9ORF72*, *VCP*, *UBQLN2*), RNA metabolism (e.g., *TARDBP*, *FUS*, *MATR3*), and cytoskeletal homeostasis (e.g., *NEK1*, *TUBA4A*, *PFN1*), cause ALS that is phenotypically indistinguishable from mutSOD1-driven disease.^28,29^ How mutations in proteins with unrelated functions converge to cause the selective death of MNs in patients is an outstanding question. The fact that all patients with ALS exhibit an accumulation of neuropathological protein aggregates suggests that impairment of protein homeostasis may be a core and unifying feature. However, the nature of this impairment remains elusive. MNs appear to be particularly susceptible to proteostasis defects,^30^ which can be triggered by a range of molecular events including the accumulation of misfolded proteins. Here, we hypothesized that mutSOD1 acts as such a trigger, impinging upon protein homeostasis upstream of pathological events including ER stress, mitochondrial dysfunction, hyperexcitability, and disruptions in the cytoskeleton.

We used SOD1-ALS patient iPSC-derived MNs to characterize the nature of proteome remodeling in response to physiologically relevant levels of mutSOD1 protein. We find that the accumulation of misfolded SOD1 protein in MNs hampers the degradation of a small panel of proteins, including valosin-containing protein (VCP). VCP plays a key role in proteasome-dependent degradation and autophagy^31,32^ and can cause rare forms of ALS itself when mutated.^33^ We show that VCP, through its ATPase activity, regulates the degradation of mutant, insoluble SOD1 protein. We also find that the VCP protein-protein interaction network is altered in mutSOD1 compared to isogenic control MNs. This “shift” in the VCP interactome is enriched for ubiquitin-related, cytoskeletal, and mitochondrial proteins. Critically, exogenous expression of VCP in mutSOD1 patient-derived MNs *in vitro* and mutSOD1 *C*. *elegans* models *in vivo* improves viability and restores impaired motility, respectively. Our work highlights a functional link between two previously unrelated ALS genes and strengthens the hypothesis that misfolded SOD1 impairs protein degradation dynamics with detrimental consequences for MN function and survival.

## RESULTS

### Mutant *SOD1* iPSC-derived patient neurons exhibit high levels of disordered soluble and insoluble SOD1 protein

**Figure 1.**
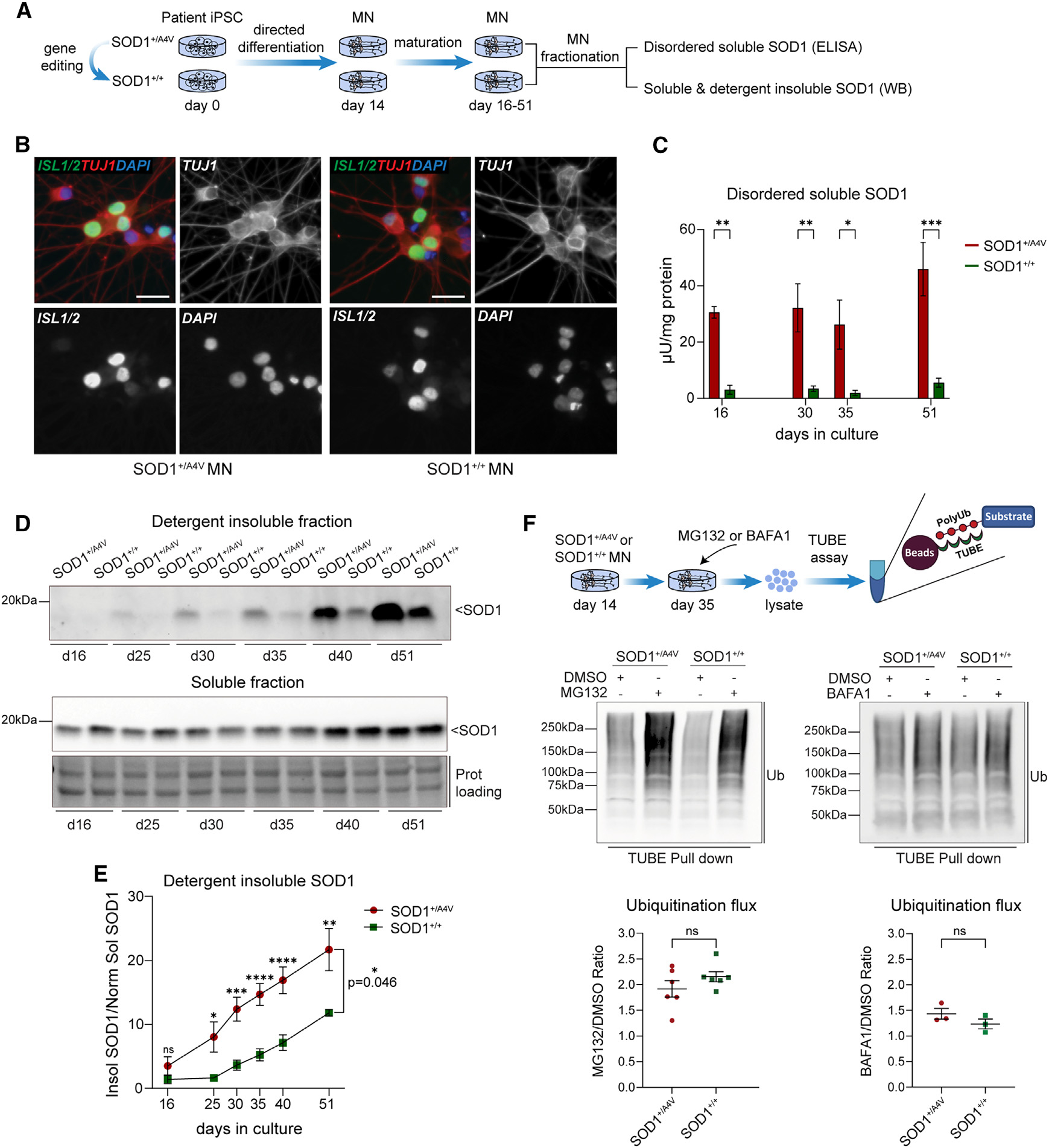
Patient neurons exhibit high levels of disordered soluble and insoluble SOD1 protein without impact on the major clearance pathways (A) Experimental schematic of MN generation and biochemical fractionation for ELISA and western blot (WB) analysis of SOD1 protein. (B) Representative images of iPSC-differentiated MNs expressing ISL1/2 and TUJ1. Scale bar, 20 μm. (C) Quantification of soluble disordered SOD1 by ELISA (n = 5 independent biological replicates, two-way ANOVA across time and genotypes, p = 0.4254; Sidak’s multiple comparisons test per time point, day 16 **p = 0.0068, day 30 **p = 0.0029, day 35 *p = 0.0215, day 51 ***p = 0.0003). (D and E) WB analysis (D) and quantification (E) of detergent-insoluble SOD1 (n = 6 independent biological replicates, two-way ANOVA across time and genotypes, *p = 0.046; Sidak’s multiple comparisons test per time point, day 16 p = 0.8352, day 25 *p = 0.0120, day 30 ***p = 0.0005, day 35 ****p < 0.0001, day 40 ****p < 0.0001, day 51 **p = 0.0022). The detergent-insoluble SOD1 is expressed relative to the normalized soluble SOD1. (F) Assessment of the ubiquitination flux in mutSOD1 and isogenic control MNs by WB. The proteasome activity was blocked with MG132 (10 μM, 8 h), and polyubiquitinated proteins were isolated from total cell lysate extracts using TUBE magnetic beads. Alternatively, the autophagosome-lysosome fusion was blocked with the inhibitor bafilomycin A1 (20 nM, 24 h), and the polyubiquitinated proteins were isolated in the same way. Representative blots (top) and quantification (bottom). Unpaired t test, for MG132 treatment, n = 6 independent biological replicates, p = 0.236; for BAFA1 treatment, n = 3 independent biological replicates, p = 0.240; ns, not significant.

To investigate the impact of mutSOD1 on proteostasis mechanisms, we first characterized the conformational state and solubility of the protein in iPSC patient-derived MNs. We used a well-characterized iPSC model consisting of a female patient-derived line harboring a deleterious *SOD1* A4V mutation and an isogenic control line in which the mutation had been corrected by genetic editing.^17^ We differentiated spinal MNs using an established 14-day protocol (Figures S1A–S1C) and collected cellular extracts at multiple time points as cells matured in culture (days 16–51 *in vitro*; Figures 1A and 1B). We then quantified the amount of disordered SOD1 protein present in the soluble protein fraction of MN extracts using a highly specific ELISA-based assay validated in patient-derived fibroblasts and iPSC-derived MNs.^34,35^ The anti-SOD1 capture antibody used in the ELISA reacts exclusively with disordered human SOD1 species and does not bind to the natively folded protein.^36^ As expected, owing to the reduced stability of mutSOD1, we found that patient MNs contained significantly higher levels of soluble disordered SOD1 relative to control neurons (Figure 1C). The amount of soluble disordered SOD1 protein was relatively similar across all time points examined but was consistently higher in patient MNs (10-fold on average relative to controls). We also evaluated the amount of total SOD1 protein that accrued in the soluble or detergent-insoluble fractions by western blot (WB). While there was no difference in total SOD1 levels within the soluble fraction (Figure S1D), mutSOD1 patient MNs exhibited a progressive accumulation of SOD1 protein in the insoluble fraction that became significantly elevated compared to the amount present in isogenic controls from day 25 onward (Figures 1D and 1E). Importantly, and in line with the intrinsic propensity for even wild-type (WT) SOD1 to misfold, a significant proportion of the WT protein progressively accumulated in the insoluble fraction even in control MNs. These analyses demonstrate that the accumulation of disordered mutSOD1 within the soluble and insoluble fractions is enhanced in patient-derived mutSOD1-expressing MNs as they age in culture.

### Mutant *SOD1* iPSC-derived patient neurons exhibit reduced degradation for specific proteins

Postmitotic cells including MNs degrade toxic polypeptides, such as disordered, damaged, or old proteins, through two major mechanisms: lysosomal and ubiquitin-proteasome-mediated degradation pathways. To investigate whether mutSOD1 compromised these pathways, we differentiated patient-derived and isogenic control MNs and assessed the ubiquitination flux using tandem ubiquitin binding entities (TUBE) after blocking the proteasome with the specific inhibitor MG132 or after blocking autophagy using the specific inhibitor bafilomycin A1 (Figure 1F). While in both cases we found significant accumulation of the ubiquitinated protein load upon blocking the pathways, this effect was not different between the two SOD1 genotypes. To supplement these experiments, we additionally quantified total ubiquitinated proteins as well as SQSTM1/p62 in mutSOD1 and isogenic control neurons by WB and confirmed a lack of substantial differences between mutSOD1 and isogenic control MNs (Figures S1E and S1F). We next assessed the level of proteasomal and lysosomal activity in MN cultures over time. We specifically used a fluorometric proteasome 20S activity assay and found no significant differences between the two genotypes up to day 51 (Figure S1G). Similarly, lysosomal function as measured by the activity of the enzyme glucocerebrosidase (GCase) did not reveal any significant alterations between mutant and control MN cultures over time (Figure S1H). These measurements suggest that mutSOD1 does not cause a global, robust impairment in protein clearance pathway activity.

**Figure 2.**
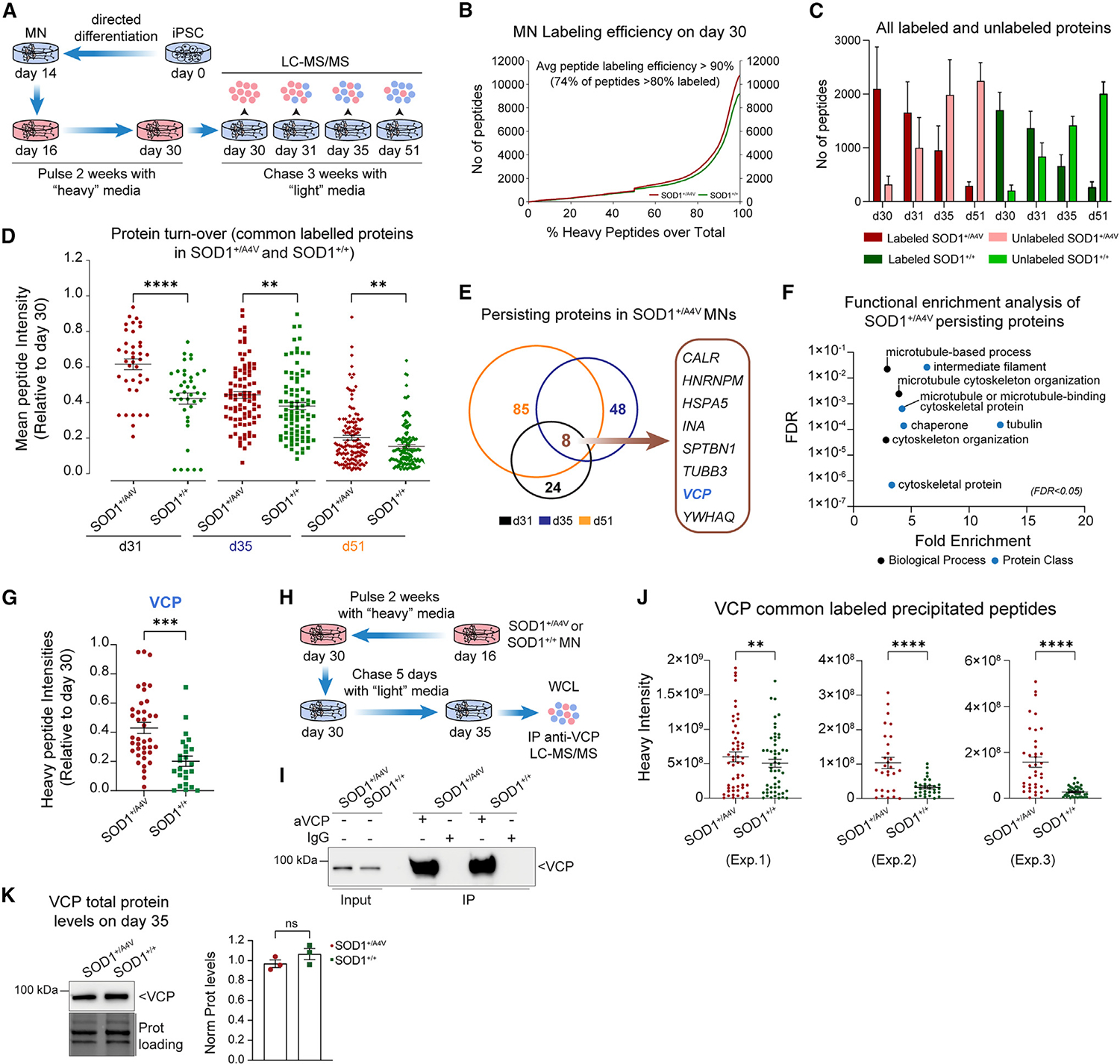
Mutant *SOD1* iPSC-derived patient neurons exhibit slower protein degradation dynamics of a small panel of proteins including VCP/p97 (A) Experimental schematic of SILAC-MS pulse-chase strategy for iPSC-MNs. (B) Assessment of labeling efficiency on day-30 MNs based on the quantification of heavy labeled peptides (lysine/arginine amino acids) over total peptides. The number of detected peptides is shown on the y axis and the percentage of heavy labeled peptides over total peptides (heavy and light) on the x axis. MN cultures with mutSOD1 are shown in red and isogenic controls in green. (C) Quantification of the total number of labeled and unlabeled peptides in both mutSOD1 and isogenic control MN cultures shown over time in culture. (D) Normalized mean peptide intensity of all labeled proteins detected in both mutSOD1 and isogenic control MNs within each one of the three time points interrogated. All values are normalized to the respective values on day 30. Each dot represents a single protein, and average and standard deviation values are shown for each time point. On day 31 (24 h after “chase”) there were 39 common proteins with a mean intensity of 0.62 and 0.42 in mutSOD1 and isogenic control MNs, respectively. On day 35 (5 days after “chase”) there were 87 common proteins with a mean intensity of 0.44 and 0.38 in mutSOD1 and isogenic control MNs, respectively. On day 51 (21 days after “chase”) there were 129 common proteins with a mean intensity of 0.20 and 0.15 in mutSOD1 and isogenic control MNs, respectively. Paired t test (two-tailed), day 31 ****p < 0.0001, day 35 **p = 0.0053, day 51 **p = 0.0012. (E) Venn diagram of the number of proteins that are more labeled (i.e., persist) in SOD1^+/A4V^ MN cultures relative to isogenic controls across all three time points (days 31, 35, and 51). The eight proteins that are more labeled across all the three time points examined are highlighted. (F) Over-representation analysis (PANTHER database) of all persisting proteins shown in (E). Based on their categorization, enriched proteins are represented in black (Biological Process) or blue (Protein Class) circles. For all the over-represented proteins, false discovery rate (FDR) < 0.05. (G) Analysis of the labeled VCP protein at the level of labeled peptides. Each dot represents a single labeled peptide on day 35, with a value normalized to the respective value of the same peptide on day 30. Unpaired t test (two-tailed), ***p = 0.0001. All data from (A) to (G) represent n = 2 independent differentiation and labeling experiments for both genotypes. (H) Schematic of the SILAC-IP LC-MS/MS experimental approach. (I) Immunoprecipitation (IP) of VPC from whole-cell lysate extracts (600 μg/reaction) derived from mutSOD1 and isogenic control MNs. IgG of the same isotype with the anti-VCP antibody was used as a negative control of the reaction. Input: 3.3%. (J) Average intensity of heavy VCP peptides that are enriched in both mutSOD1 and isogenic control MN cultures on day 35 upon immunoprecipitation. The comparison is made between identical (common) VCP peptides in both genotypes. Paired t test, Wilcoxon correction, n = 3 independent differentiations; experiment 1, **p = 0.0024; experiment 2, ****p < 0.0001; experiment 3, ****p < 0.0001. (K) Total VCP protein levels under baseline conditions on day 35 in patient and isogenic control MNs. Unpaired t test (two-tailed), n = 3 independent differentiations, p = 0.228; ns, not significant.

We next investigated the impact of mutSOD1 on protein degradation by a more granular approach. We specifically differentiated equal amounts of mutSOD1 and isogenic control MNs (Figures S1B and S1C) and used stable isotope labeling with amino acids in cell culture (SILAC) in combination with liquid chromatography-tandem mass spectrometry (LC-MS/MS)-based proteomic analysis to examine the rate of degradation of individual proteins in a “pulse-chase” paradigm (Figure 2A). After 2 weeks of culturing MNs with medium containing exclusively lysine/arginine amino acids highly enriched with “heavy” stable isotopes (“pulse”), we found that most of the peptides were successfully labeled (i.e., >80%) across two independent experiments and two genotypes (Figure 2B). We then switched to the “light” culture medium, which contained non-labeled amino acids, and collected whole-cell extracts of MNs for MS analysis after 1 day (day 31 *in vitro*), 5 days (day 35 *in vitro*), and 21 days (day 51 *in vitro*) of “chase.” In two independent experiments we quantified—on average—more than 1,300 labeled proteins containing “heavy” amino acids. As proteins gradually degraded over time in culture, we found that the levels of the “heavy” labeled (i.e., older) proteins decreased in both genotypes, while at the same time the number of newly synthesized unlabeled proteins (and total peptides) increased (Figures 2C and S2A). At least 50% of labeled peptides were degraded within 5 days, while a small fraction (approximately 15%) remained labeled even after 21 days of “chase” in both genotypes. These “heavy” labeled or persisting proteins (Table S1), which represent the longer-lived proteins in postmitotic human MNs within the time frame of this experiment, were localized in various cellular compartments (Figure S2E) and were enriched for cytoskeletal proteins, tubulins, and chaperones as assessed by gene ontology (GO) analysis using the PANTHER functional annotation tool (Figure S2F and Table S1). Intriguingly, within this group we detected several ALS-associated proteins including KIF5A, FUS, VCP, DCTN1, MATR3, SFPQ, TUBA4A, PRPH, and STMN2 (Figure S2E), suggesting that long-lived proteins (or proteins with slower degradation kinetics) may be contributing to the vulnerability of MNs to ALS disease mechanisms.

To confirm the ability of our SILAC-based “pulse-chase” proteomic analysis method to identify accumulated proteins, we repeated the labeling paradigm for 2 weeks and subsequently treated isogenic control MN cultures with the proteasomal inhibitor MG132 (1 μM, 24 h) (Figure S2B). Analysis of the proteome by LC-MS/MS in these cultures showed that blocking the proteasome indeed caused a highly significant accumulation of peptides with heavy intensities as well as all peptides assessed by both heavy and light intensities (Figure S2C), further validating this experimental paradigm.

To investigate the hypothesis that the continuous production or impaired clearance of mutSOD1 might hinder the degradation of specific proteins, we next cataloged all proteins commonly identified in both mutSOD1 and isogenic control MN datasets at specific time points (39, 87, and 129 proteins 1, 5, and 21 days post chase, respectively) and examined their level of “heavy” old protein remaining (Figure 2D and Table S2). We found that relative to day 30, there was a significantly higher mean MS1 peak intensity of the “heavy” labeled peptides in patient MNs across all time points, with the overwhelming majority of proteins being more labeled (or persisting) in patient MN extracts: specifically, 82%, 64%, and 72% after 1, 5, and 21 days post chase, respectively (Figures 2D and 2E). To identify over-represented classes within the proteins that persist in patient MNs, we performed GO analysis that revealed significant enrichment for “cytoskeletal proteins,” “chaperones,” and “microtubule or microtubule-binding cytoskeletal proteins” and associated biological processes (Figure 2F and Table S2). Interestingly, eight proteins were found to persist in mutSOD1 MNs across all the time points inspected (Figure 2E). These included: TUBB3, which encodes the major neuronal form of tubulin; the heat-shock protein HSPA5, which plays a fundamental role in protein folding within the ER; SPTBN1, a ubiquitously expressed β-spectrin that facilitates membrane scaffolding; the RNA splicing factor hnRNPM; the intermediate filament internexin-alpha (INA); the multifunctional ER protein calreticulin; the signal transduction protein YWHAQ; and VCP/p97. More stringent analysis of the turnover of these eight proteins at the level of single peptides revealed that SPTBN1 and VCP were significantly more labeled in mutSOD1 relative to isogenic control MNs (Figures 2G and S2D). VCP stands out, as it plays a prominent role in both the ubiquitin-proteasome system (UPS) and in autophagy-dependent protein degradation mechanisms,^37–39^ and importantly rare genetic mutations in the *VCP* gene can cause familial ALS.^33^ Collectively these experiments demonstrate that while there is no evidence for a global defect in protein degradation flux, the accumulation of mutSOD1 in patient MNs affects the degradation rate of a relatively small and specific subset of proteins, including VCP.

### A pool of VCP persists in mutSOD1 iPSC-derived MNs

To validate the persistence of VCP in mutSOD1 MNs, we immunoprecipitated endogenous VCP protein from three rounds of independently differentiated and pulse-chased SILAC MN extracts and again performed LC-MS/MS-based proteomic analysis to specifically and accurately quantify VCP peptides (Figures 2H and 2I). We performed the immunoprecipitation (IP) on extracts from days 30 and 35 *in vitro*, i.e., 0 and 5 days after the beginning of the “chase” period. At this time point we found significantly higher levels of the “heavy” labeled (i.e., old or persisting) VCP protein in mutSOD1 relative to control MNs in our original, unbiased MS analysis (Figures S2G and 2G). The VCP-enriched IP fractions were subjected to quantitative MS, and comparison of identical VCP peptides between the patient and control MNs showed that “heavy” labeled VCP was consistently more abundant in mutSOD1 MNs, confirming our initial observation (Figure 2J). Remarkably, the robust persistence of “older” VCP in mutSOD1 MNs did not result in an increase in total steady-state protein level (Figures 2K and S2H), nor did it result in the accumulation of VCP within the insoluble fraction (Figure S2I).

### The SOD1 A4V mutation is sufficient to alter the protein degradation dynamics of VCP in MNs

**Figure 3.**
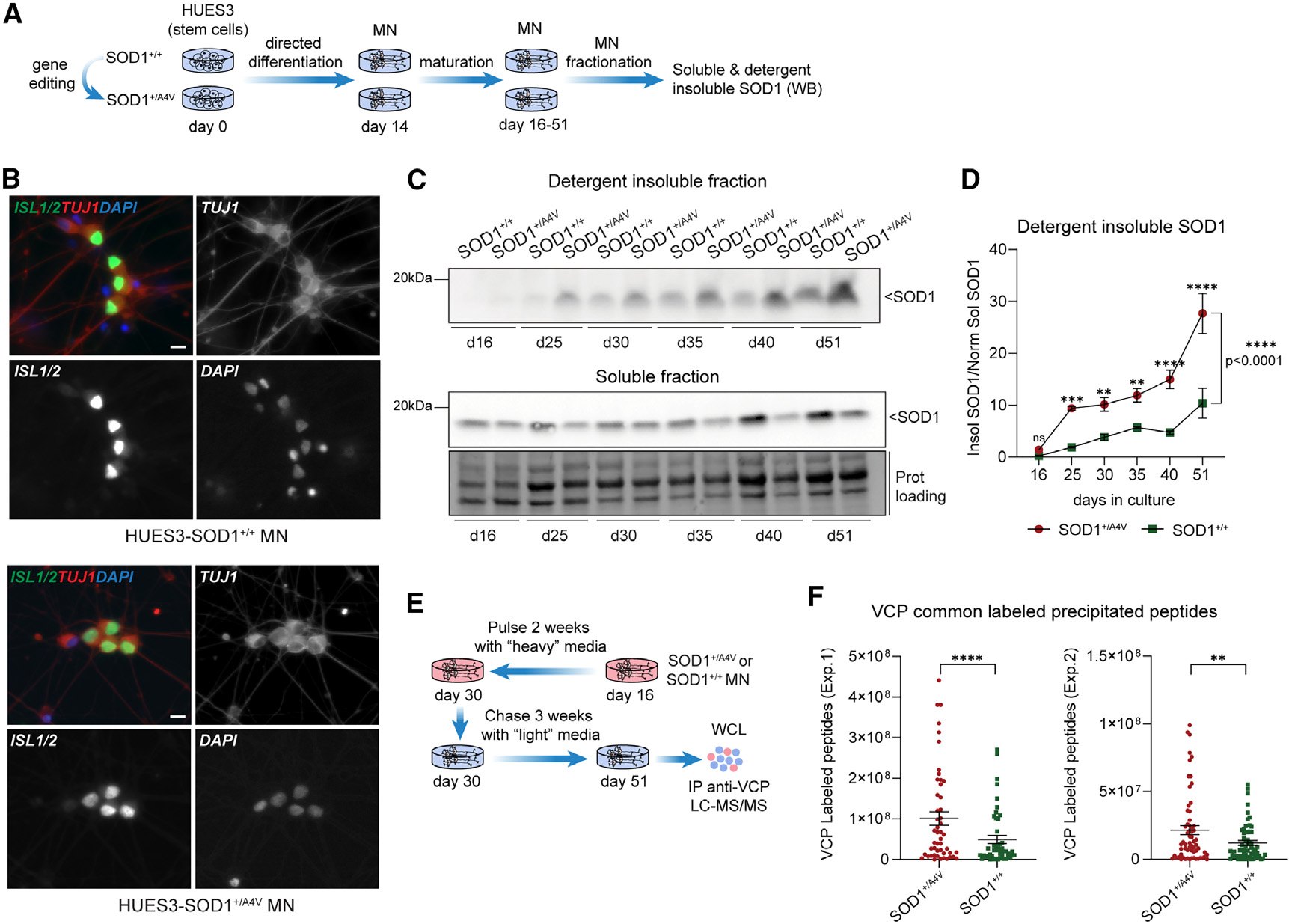
The *SOD1* A4V mutation is sufficient to alter VCP turnover (A) Experimental schematic of the HUES3 stem cell editing and MN-directed differentiation. (B) Representative images of stem-cell-differentiated MNs expressing ISL1/2 and TUJ1 on day 25. Scale bar, 10 μm. (C and D) WB analysis (C) and quantification (D) of the detergent-insoluble SOD1 levels in HUES3-SOD1^+/+^ or HUES3-SOD1^+/A4V^ MNs across time. The detergent-insoluble SOD1 (top blot) is expressed relative to the normalized soluble SOD1 (bottom blot). Two-way ANOVA across time and genotypes, ****p < 0.0001; Sidak’s multiple comparisons test per time point, day 16 p = 0.9571, day 25 ***p = 0.0008, day 30 **p = 0.0033, day 35 **p = 0.0037, day 40 ****p < 0.0001, day 51 ****p < 0.0001; ns, not significant; n = 3 independent differentiations. (E) Schematic representation of the SILAC-IP LC-MS/MS approach. (F) Average intensity of heavy VCP peptides that are enriched in both HUES3-SOD1^+/+^ and HUES3-SOD1^+/A4V^ MN cultures on day 51 upon immunoprecipitation. The comparison is done between identical, common VCP peptides in both genotypes. Paired t test, Wilcoxon correction; n = 2 independent differentiations; experiment 1, ****p < 0.0001; experiment 2, **p = 0.009.

To investigate whether the *SOD1* A4V mutation is sufficient to induce the alteration of VCP degradation dynamics, we next used a set of isogenic stem cell lines where the A4V mutation was engineered within a control genetic background of a human embryonic stem cell line (lines HUES3 and HUES3-SOD1A4V; Figure 3A).^40^ Following the same directed differentiation protocol, we generated and characterized spinal MN cultures from both mutSOD1 and isogenic control lines (Figures 3B, S3B, and S3C). We first investigated the progressive solubility of SOD1 protein by quantifying soluble and insoluble protein across 51 days *in vitro* (Figure 3C). We observed gradual accumulation of SOD1 protein within the detergent-insoluble fraction that was significantly higher for the mutant MNs, with the maximum difference on days 40–51 (Figure 3D). Conversely, the amount of soluble SOD1 protein became significantly lower in mutant MNs on day 51 (Figure S3C). These analyses demonstrate that mutSOD1 accumulates within the insoluble fraction in a similar pattern but with slightly delayed dynamics in these independent cell lines. To investigate the degradation of VCP in HUES3-SOD1A4V MNs, we repeated the pulse-chase SILAC labeling paradigm followed by IP and LC-MS/MS (Figure 3E). Comparison of the identical VCP peptides that were precipitated from whole-cell extracts of mutSOD1 and isogenic control MNs revealed a robust and highly significant abundance of older VCP in HUES3-SOD1A4V MNs (Figure 3F). These results confirm our initial findings and additionally demonstrate that the *SOD1* A4V mutation is sufficient to induce the biochemical change in SOD1 protein and the dependent shift in the degradation dynamics of VCP, and other potential genetic variants in the background of the patient MNs do not account for this phenotype.

### VCP accumulates in postmortem ALS-SOD1 patient tissue

**Figure 4.**
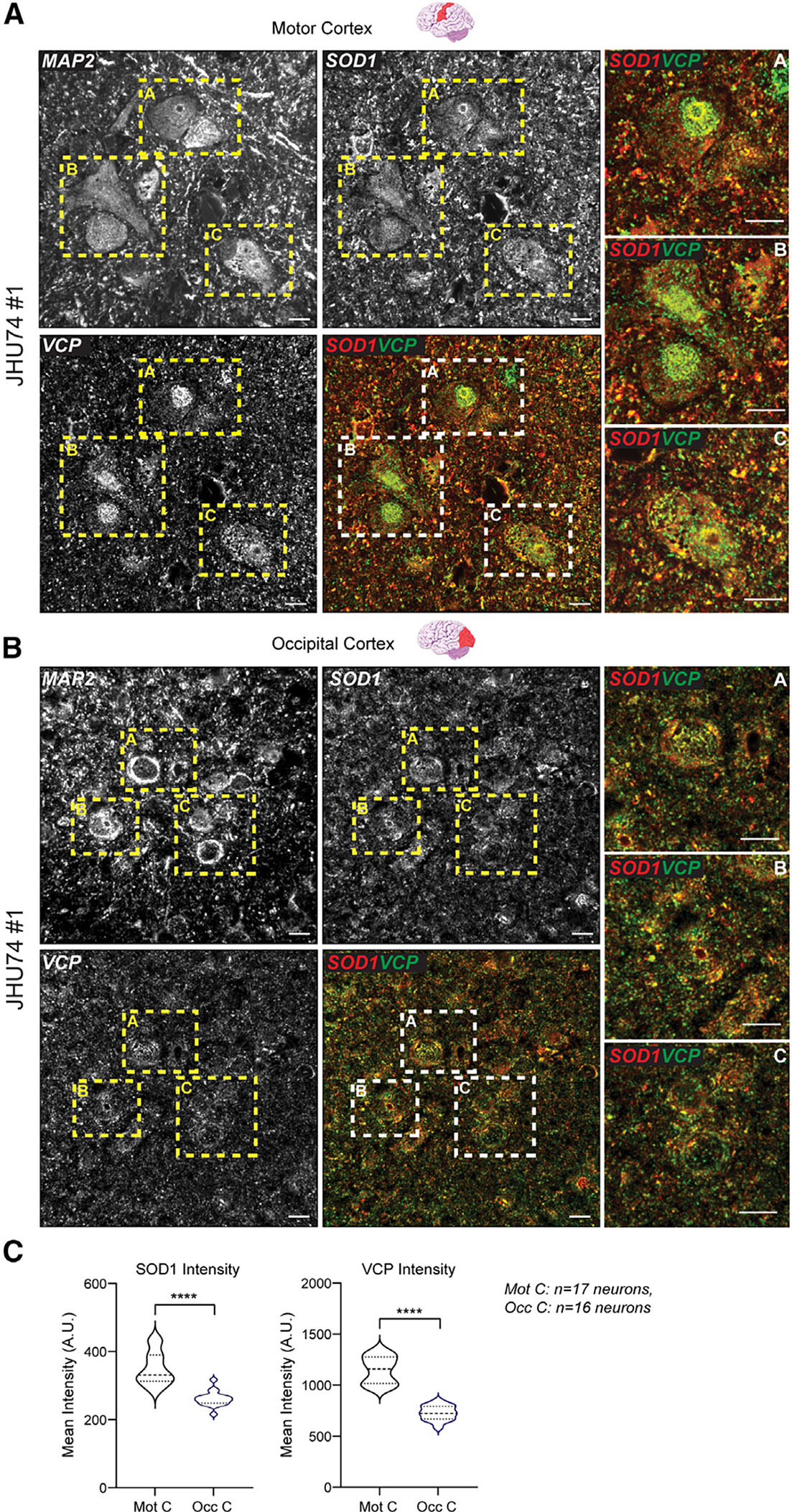
Accumulated VCP in postmortem tissue of an SOD1^+/A4V^ ALS patient (A and B) Immunohistochemistry of VCP and SOD1 in postmortem motor (affected) (A) and occipital (unaffected) (B) cortex from an ALS patient (patient #1-JHU74) carrying the A4V mutation in the *SOD1* gene. The pictures on the right column represent the magnified regions within the yellow dashed squares. Scale bar, 20 μm. (C) Quantification of SOD1 and VCP intensities in MAP2^+^ neurons within the motor or occipital cortex. Motor cortex, n = 17 neurons; occipital cortex, n = 16 neurons. Unpaired t test (two-tailed), SOD1 p < 0.0001; VCP p < 0.0001.

To determine whether the slower turnover of VCP in mutSOD1 iPSC-derived MNs *in vitro* is relevant to patient pathology, we next examined postmortem ALS mutSOD1 patient tissue. We specifically acquired motor cortex and occipital cortex, two brain regions that are affected and non-affected in ALS disease, respectively, from two patients harboring an *SOD1* A4V mutation. Immunohistochemistry (IHC) analysis showed that in both patients examined, mutSOD1 and VCP exhibited overlapping accumulation within large MAP2^+^ cortical neurons within the affected motor cortex but not in the occipital cortex (Figures 4 and S4). Critically, quantitative analysis showed that both SOD1 and VCP accumulated at significantly higher levels within neurons in the affected motor cortex relative to neurons in the unaffected occipital cortex across both patients (Figures 4C and S4C). These findings suggest that VCP homeostasis is differentially affected between mutSOD1-vulnerable and invulnerable patient brain tissue.

### VCP exhibits an altered interactome in mutant *SOD1* iPSC-derived MNs

**Figure 5.**
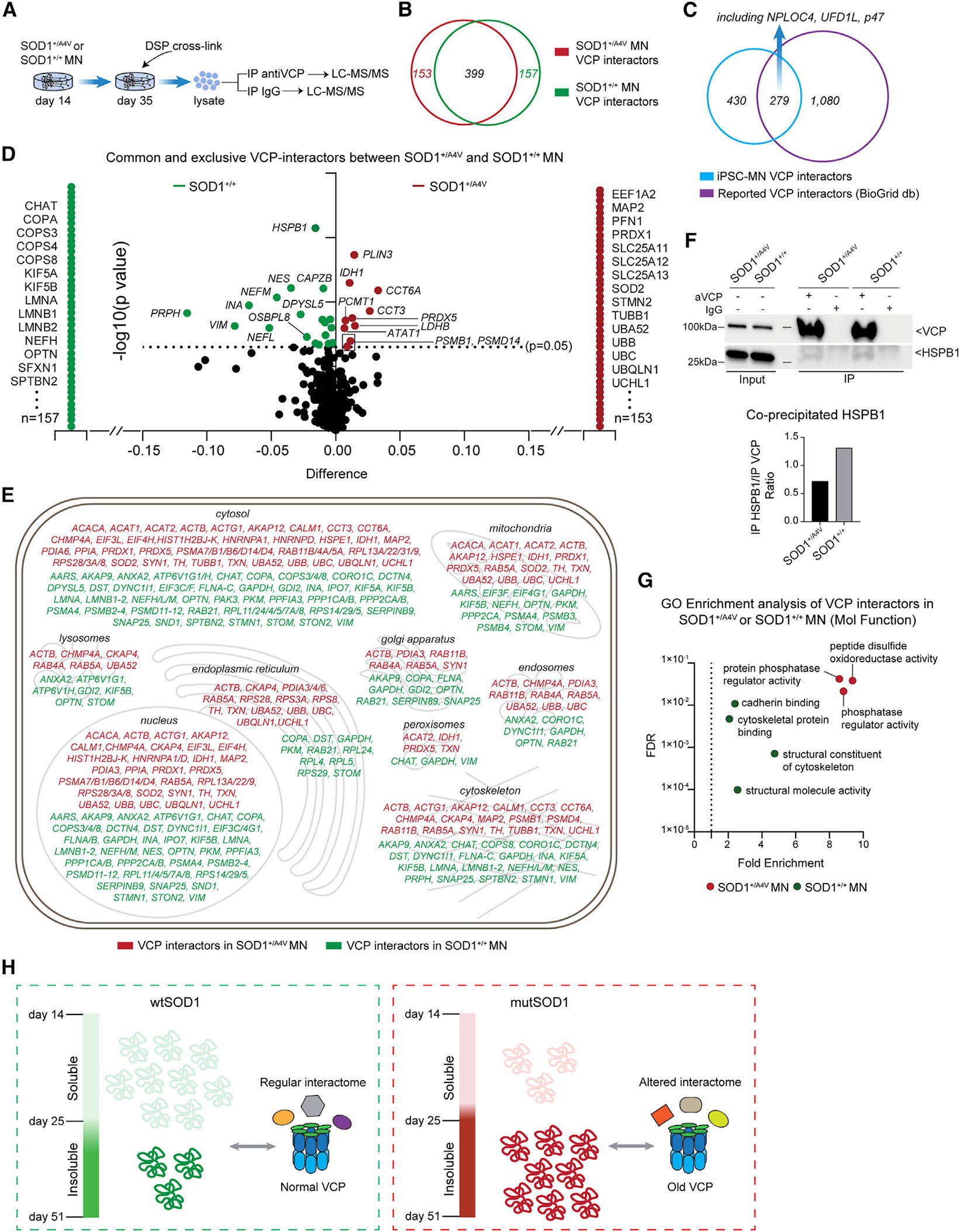
The interactome of VCP alters in patient MNs (A) Schematic representation of the crosslink/IP/MS experimental workflow. The MNs on day 35 were crosslinked with the reversible crosslinker DSP (1 mM) for 20 min before they were collected. The cell extracts were subjected to immunoprecipitation (IP) using a specific antibody against VCP, and the precipitates were further analyzed by LC-MS/MS. (B and C) Venn diagrams showing the distribution of VCP co-precipitated proteins between the patient or isogenic control MNs (B) and their overlap with the reported (known from the literature or predicted) VCP interactors in the BioGrid database (C). (D) Volcano plot of the 399 shared VCP interactors between the genotypes. The red dots on the right and the green dots on the left represent VCP interactors that are enriched in either of the two genotypes. The red and green columns represent some of the exclusive VCP interactors in patient or isogenic control MNs, respectively. (E) Subcellular localization of some of the exclusive or enriched VCP interactors in either SOD1^+/A4V^ (red) or SOD1^+/+^ (green) MNs. (F) Validation by IP/WB of HSPB1 as an enriched interactor of VCP in isogenic control MNs. The levels of co-precipitated HSPB1 were normalized to the respective level of precipitated VCP. (G) GO analysis (WebGestalt web tool) of the over-represented groups of the VCP interactome in either SOD1^+/A4V^ (red) or SOD1^+/+^ (green) MNs. FDR < 0.05. (H) Schematic representation of our working model. Mutant SOD1 protein progressively becomes more insoluble than WT protein (red and green panels, respectively) and is associated with the slower turnover of VCP, which exhibits both gain- and loss-of-function interactions in mutSOD1 MNs.

VCP/p97 is a conserved ATPase that facilitates the degradation of numerous protein substrates by ubiquitin-dependent mechanisms, primarily acting through the proteasome.^39,41^ As such, it plays a critical role in diverse cellular functions, and its persistence could have major implications for the homeostasis of mutSOD1 MNs. The interactions of VCP with other adaptor proteins are exceptionally dynamic, while it is well established that disease-causing mutations in *VCP* can alter the binding to and processing of substrate proteins.^42,43^ To better understand how the accumulation of the pool of “older” VCP could impact its function, we next sought to examine its interacting adaptor and substrate proteins in mutSOD1 and isogenic control MNs. Because the interactions of VCP are highly dynamic and consequently difficult to capture, we used the chemical crosslinker DSP (dithiobis(succinimidyl propionate)) to stabilize physiological VCP complexes before collecting and lysing cells for IP and LC-MS/MS analysis (Figure 5A). We performed three independent IP experiments on day 35 using a VCP antibody or immunoglobulin G (IgG) as a negative control and identified 709 proteins that were enriched within the VCP fraction. Of these, 153 were identified exclusively within mutSOD1 patient MNs and 157 were identified exclusively within control MNs (Figure 5B). Critically, almost 40% of proteins (279 out of 709) were previously reported as VCP-interacting proteins based on the BioGrid database (Figure 5C), a considerable overlap considering that our experiments represent the first attempt of identifying VCP substrates in postmitotic human neurons. Additionally, within this list of proteins we detected several known co-factors of VCP such as NPLOC4, UFD1L, and NSFL1C (P47). GO analysis of the shared VCP interactors between both SOD1 genotypes revealed enrichment of cytoskeletal proteins and metabolic processes, suggesting that VCP is associated with the recycling of structural and metabolic components in MNs (Figure S5A).

To address how mutSOD1 might be affecting VCP we next focused on differentially interacting proteins, which encompassed several new interactions as well as loss of interactions within mutSOD1 MNs (Figures 5D and 5E; Table S3). Notable loss-of-function interactors include structural and functional components of the cytoskeleton such as neuronal filament-related proteins (e.g., NEFH, NEFM, NEFL, INA), kinesins that transport organelles through neurites and axons (e.g., KIF5A, KIF5B), other cytoskeletal proteins such as PRPH and SPTBN2, and chaperones including the small heat-shock protein HSPB1, which we validated in independently differentiated MN samples by IP and WB (Figure 5F). Conversely, gain-of-function VCP interactions within mutSOD1 MNs were enriched for proteins associated with enzymatic activities (e.g., peptide disulfide oxidoreductase activity, phosphatase regulator activity) and several ubiquitin-related proteins (e.g., UBB, UBC, UBA52, UBQLN1), as well as mitochondrial proteins (e.g., PRDX1, PRDX5, SOD2) and STMN2, which was recently identified as a TDP-43 target RNA that becomes mis-spliced and downregulated in non-SOD1 patients with ALS^44,45^ (Table S3 and Figure 5G). Collectively, these findings suggest that the progressive accumulation of insoluble mutSOD1 disrupts VCP activity by causing a shift in the classes of its protein substrates (Figure 5H).

### VCP modulates the accumulation of detergent-insoluble mutSOD1 protein

**Figure 6.**
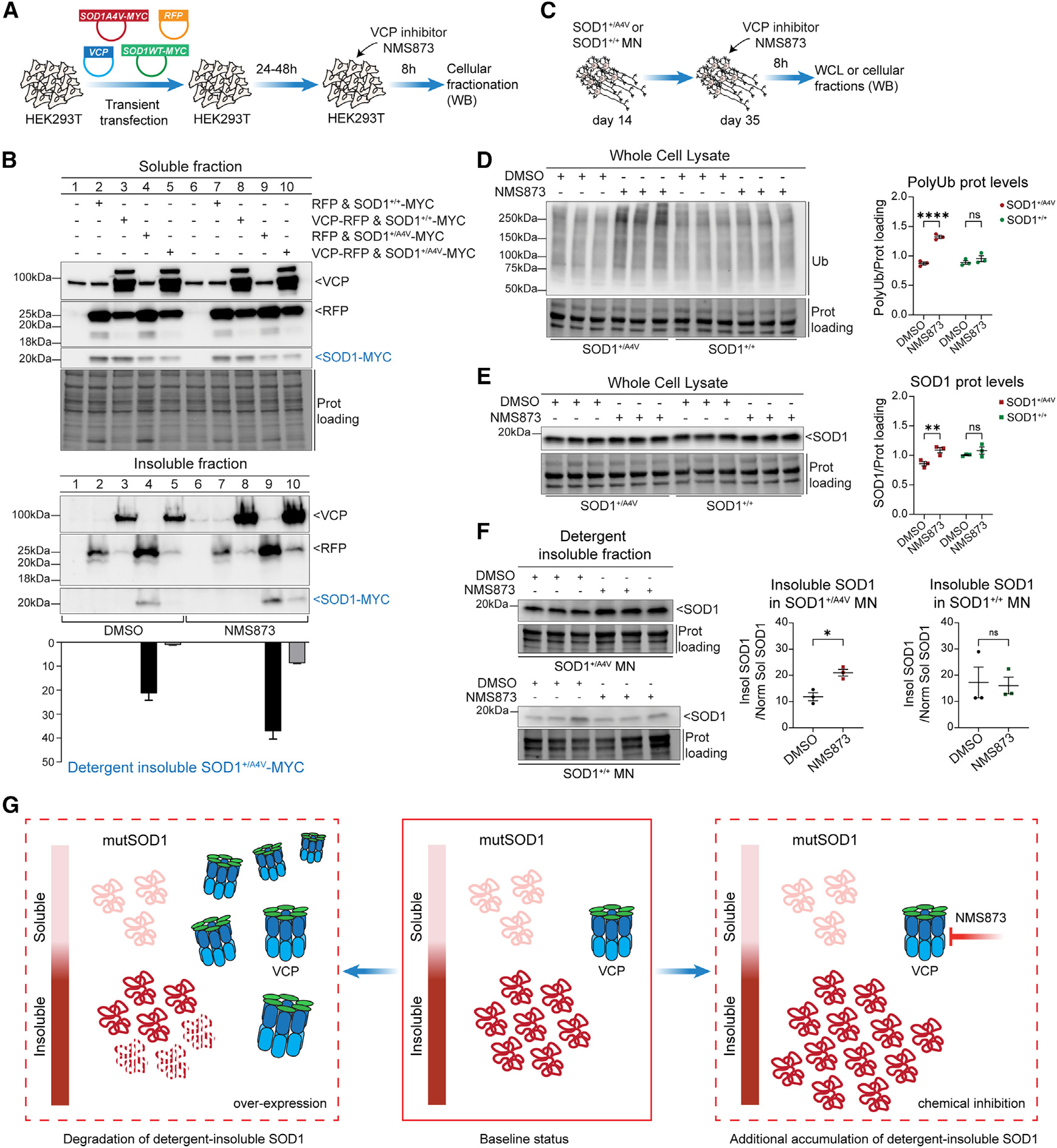
VCP impacts the solubility of SOD1 (A) Experimental workflow of HEK293T transfection and treatment. HEK293T cells were transfected with SOD1WT-MYC or SOD1A4V-MYC plasmids in combination with VCP-RFP or RFP plasmids. The cells were incubated up to 48 h and treated with the allosteric VCP inhibitor NMS873 (10 μM, 8 h). The cells were lysed and subjected to fractionation for biochemical analysis. (B) WB analysis of HEK293T-transfected cells. Lanes 1–5 correspond to DMSO-treated cells and lanes 6–10 to NMS873-treated cells. The bar graph corresponds to the levels of the detergent-insoluble SOD1-MYC (insoluble fraction, bottom blot). The detergent-insoluble SOD1-MYC is normalized to the soluble SOD1-MYC levels (soluble fraction, top blot); n = 2 independent transfections. (C) Schematic representation of patient or isogenic control MNs treated with NMS873 (10 μM, 8 h) on day 35 and lysed for biochemical analysis. (D and E) WB analysis and quantification of whole-cell extracts from DMSO- or NMS873-treated MNs and quantification of poly-Ub (D) or SOD1 (E) protein levels. For poly-Ub, two-way ANOVA (treatment × genotype), *p = 0.0152; Sidak’s multiple comparisons test per treatment, SOD1^+/A4V^ MN ****p < 0.0001, SOD1^+/+^ MN p = 0.396. For SOD1 two-way ANOVA (treatment × genotype), p = 0.2170; Sidak’s multiple comparisons test per treatment, SOD1^+/A4V^ MN **p = 0.0099, SOD1^+/+^ MN p = 0.4156; ns, not significant; n = 3 biological replicates. (F) WB analysis of detergent-insoluble SOD1 levels in SOD1^+/A4V^ or SOD1^+/+^ MNs upon treatment with NMS873. Unpaired t test (two-tailed), SOD1^+/A4V^ MN *p = 0.0105, SOD1^+/+^ MN p = 0.8696; ns, not significant; n = 3 biological replicates. (G) Impact of VCP on SOD1 accumulation. When VCP is overexpressed (left) in the context of mutSOD1, the levels of detergent-insoluble SOD1 protein are decreased. In contrast, upon chemical inhibition of VCP with NMS873 (right), there is significant accumulation of SOD1 protein within the detergent-insoluble fraction.

Although we did not identify SOD1 in any of the VCP immunoprecipitated material, we next sought to determine whether VCP activity has the capacity to modulate the degradation of mutSOD1 protein indirectly. We first used a heterologous expression cell model to co-transfect WT or mutSOD1 A4V protein with or without VCP or RFP as a control. As before, we biochemically purified cell extracts into soluble and detergent-insoluble fractions and analyzed the abundance of SOD1 by WB (Figure 6A). We observed that mutant but not WT SOD1 accumulated in the detergent-insoluble fraction and that co-expression of VCP dramatically diminished this accumulation (Figure 6B, lanes 1–5). Notably, VCP did not affect WT SOD1 protein, nor did it result in greater abundance of mutSOD1 within the soluble fraction, suggesting that it mediates the selective degradation of mutant disordered SOD1 protein (Figure 6B, lanes 1–5). To determine whether this effect was dependent on the ATPase activity of VCP, we repeated these experiments with the use of the allosteric VCP inhibitor NMS873 (10 μM, 8 h) and found that this treatment reduced its degradation capacity on mutSOD1 protein (Figure 6B, lanes 5 and 10). Intriguingly, use of the NMS873 inhibitor alone also caused an increased accumulation of mutSOD1 in the insoluble fraction, likely on account of blocking endogenous VCP activity, further demonstrating that the clearance of mutSOD1 is dependent on VCP activity (Figure 6B, lanes 4 and 9).

We next examined the interplay between VCP activity and SOD1 in iPSC-derived MNs (Figure 6C). Treating day-35 MN cultures from both pairs of stem cell lines with the allosteric VCP inhibitor NMS873 caused a small but highly significant increase in total SOD1 levels in the case of mutant protein, while the WT was unaffected (Figures 6E and S6C). Additionally, blocking VCP activity caused a dramatic accumulation of total ubiquitinated proteins exclusively in the mutSOD1 MN cultures (Figure 6D), suggesting that VCP plays a crucial role in protein degradation in the context of the *SOD1* A4V mutation but not in control neurons. Lastly, quantification of SOD1 levels upon enzymatic inhibition of VCP and cellular fractionation revealed that SOD1 selectively accumulated within the detergent-insoluble fraction of patient MNs (Figures 6F, S6A, and S6B). Collectively, these experiments in heterologous expression cells and patient MNs showcase that VCP plays a prominent role in the regulation of SOD1 solubility and degradation (Figure 6G).

### VCP ameliorates mutSOD1 toxicity in iPSC-MNs *in vitro* and *C*. *elegans* models *in vivo*

**Figure 7.**
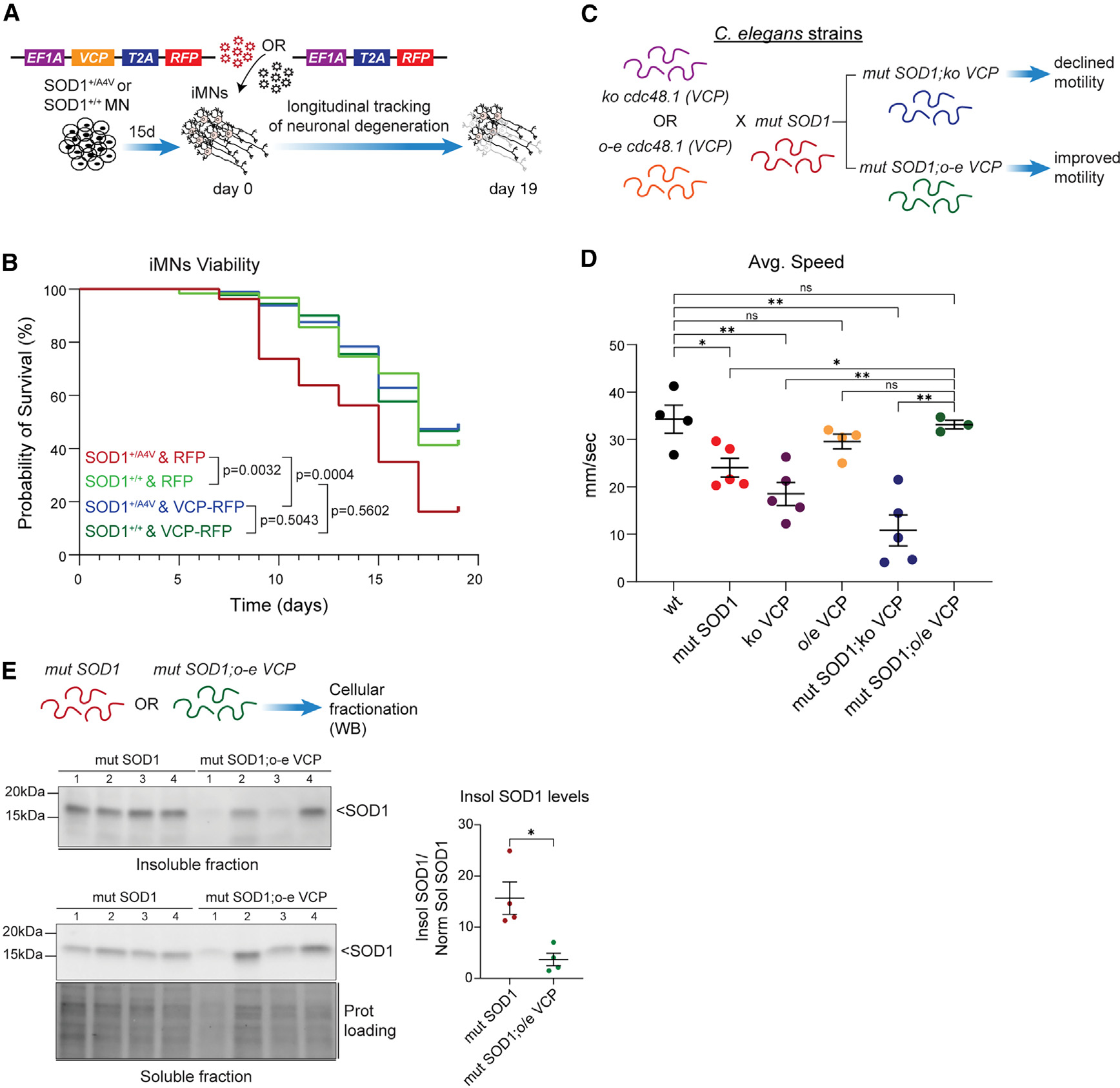
VCP ameliorates mutSOD1 toxicity in iPSC-MNs *in vitro* and *C. elegans* models *in vivo* (A) Experimental schematic of MN viability assay. MNs were differentiated from mutSOD1 and isogenic control iPSCs and infected with either *LV-VCP-T2A-RFP* or *LV-RFP* to monitor progressive degeneration by longitudinal time-lapse imaging microscopy. (B) Probability of survival of mutSOD1 and isogenic control iMNs upon VCP-RFP or RFP expression across 3 weeks in culture; n = 1 differentiation and 3 technical replicates; for SOD1^+/A4V^ iMN *LV-VCP-T2A-RFP* n = 220, *LV-RFP* n = 197; for SOD1^+/+^ iMN *LV-VCP-T2A-RFP* n = 200, *LV-RFP* n = 198; Gehan-Breslow-Wilcoxon test. (C) Experimental schematic of genetic interaction experiments with *C*. *elegans* strains expressing human mutSOD1 protein (*IW8*), with overexpression (o-e) and knockout (ko) of the VCP ortholog *cdc48*.*1*. (D) Quantification of the average speed per second of *C*. *elegans* strains examined. One-way ANOVA for genotype, p < 0.0001; Unpaired t test to compare individual genotypes: (1) WT vs. mutSOD1 *p = 0.0206, (2) WT vs. ko VCP **p = 0.0042, (3) WT vs. o-e VCP p = 0.2096, (4) WT vs. mutSOD1; ko VCP **p = 0.0013, (5) WT vs. mutSOD1; o-e VCP p = 0.7650, (6) mutSOD1; o-e VCP vs. mutSOD1; ko VCP **p = 0.0023, (7) mutSOD1; o-e VCP vs. o-e VCP p = 0.1325, (8) mutSOD1; o-e VCP vs. ko VCP **p = 0.0044, (9) mutSOD1; o-e VCP vs. mutSOD1 *p = 0.0154; n = 3–5 independent experiments, n = 5–10 worms per experiment. (E) Quantification of SOD1 protein within the insoluble and soluble fractions in mutSOD1 worms and mutSOD1 overexpressing VCP. The amount of detergent-insoluble SOD1 is expressed relative to the amount of normalized soluble SOD1. Unpaired t test (two-tailed), *p = 0.0123; n = 4 independent preparations of worm cultures.

Having established that VCP homeostasis is altered in mutSOD1 MNs and that VCP modulates the degradation of mutant insoluble SOD1 protein, we next wondered whether modulating VCP expression would affect mutSOD1 toxicity. To assess this, we generated induced motor neurons (iMNs) from SOD1^+/A4V^ patient, isogenic, and non-disease control iPSCs lines^46^ and infected them with lentivirus expressing either VCP-T2A-RFP or RFP alone as a negative control (Figure 7A). Analysis of VCP-RFP levels showed that mutant and isogenic control lines were infected to a similar degree (Figure S7A). We then monitored individual neurons using automated microscopy and longitudinal tracking across time in culture. We found that, as expected, mutSOD1 iMNs degenerated faster relative to their isogenic controls, while expression of VCP significantly increased the probability of MN survival in the context of an SOD1 A4V mutation. In contrast, the survival of neurons generated from the isogenic control iPSC line, or an unrelated non-disease control line, was unaffected by VCP expression (Figures 7B and S7B–S7D).

Lastly, to determine whether there is crosstalk between mutSOD1 and VCP in an intact nervous system *in vivo*, we used an established *C*. *elegans* ALS model for mutSOD1 toxicity.^47–49^ Worms expressing mutSOD1 in the nervous system display a significant locomotor deficit as measured by reduced speed in liquid compared to WT animals (Figures 7C and S7E). While humans have a single VCP/p97 protein, worms have a pair of orthologs termed CDC-48.1 and CDC-48.2.^50^ We found that worms with a putative null allele of *cdc-48*.*1* also exhibit a significant decline in average speed, while in contrast worms overexpressing *cdc-48*.*1* are indistinguishable from WT animals (Figure 7D). We generated worms with a deletion of *cdc-48*.*1* within the mutSOD1 background and found that loss of CDC-48.1 significantly exacerbated the locomotor deficit incurred by mutSOD1 (Figure 7D). Critically, overexpression of CDC-48.1 protein within the mutSOD1 background significantly ameliorated the observed speed defect, resulting in worms that were indistinguishable from their WT counterparts (Figure 7D). To investigate whether overexpression of CDC-48.1 ameliorated mutSOD1 toxicity by modulating the levels of insoluble SOD1 protein, we performed biochemical fractionation coupled to WB using an adapted lysis method previously optimized for mutSOD1 *C*. *elegans* models (Figure 7E).^47^ This analysis showed that the levels of detergent-insoluble SOD1 were significantly reduced in *mutSOD1*;*o-e cdc-48*.*1* compared to *mutSOD1* strains, highlighting the role of the VCP ortholog in the clearance of accumulated SOD1 *in vivo*. Collectively, these experiments demonstrate that exogenous expression of VCP in human iPSC-MNs *in vitro* and *C*. *elegans* models *in vivo* suppresses the toxic effects of mutSOD1.

## DISCUSSION

Proteome homeostasis is regulated by factors involved in protein production, processing, and degradation.^30,51,52^ It has long been postulated that the accumulation of misfolded mutSOD1 protein can disrupt this finely controlled balance to cause MN dysfunction and eventual degeneration.^30,51^ In this study, we characterized the proteome-wide degradation dynamics of patient MNs harboring an *SOD1* mutation that contain high levels of soluble and progressively accumulating insoluble mutSOD1 protein. We identified several proteins that exhibited slower turnover, which were predominantly associated with protein folding and cytoskeletal homeostasis. Among them was VCP, which plays a prominent role in ubiquitin-based degradation and can cause rare forms of ALS.^33^ We showed that VCP accumulates with mutSOD1 in the affected motor cortex of ALS patients and exhibits a different interactome in mutSOD1 neurons, while exogenous expression of VCP within *in vitro* and *in vivo* disease models alleviated mutSOD1 toxicity. These effects were in part due to the ability of VCP to modulate the degradation of insoluble mutSOD1 protein. Our work suggests that alterations in VCP homeostasis in the context of mutSOD1 protein may be one of the primary hubs controlling downstream degenerative pathways in patient MNs.

VCP is a ubiquitously expressed ATPase that affects cellular homeostasis through its primary function in protein quality control.^39,41^ Energy generated by hydrolysis of ATP allows VCP to change its conformation and extract ubiquitinated protein substrates for degradation by the UPS.^53^ The diverse functions of VCP are facilitated by several co-factors that form adaptor complexes and mediate ubiquitin-dependent degradation processes including ER-associated degradation (ERAD) of misfolded or old and damaged proteins, degradation of polypeptides through the endosomal and lysosomal pathways, and the clearance of cytosolic aggregates through the autophagy pathway.^32,38,54–56^

The critical role of VCP in cellular health is highlighted by the fact that genetic mutations in *VCP* have been associated with multiple neurodegenerative diseases including ALS,^33^ inclusion body myopathy associated with Paget disease of bone and frontotemporal dementia,^57^ and Huntington’s disease.^58^ The precise mechanisms by which mutations trigger pathogenesis in the context of these diverse and devastating diseases remain unclear. Autosomal dominant mutations in *VCP* account for up to 1% of all ALS cases and, while the exact cause of MN dysfunction in these patients is unknown, mutations have been associated with both loss-of-function and gain-of-function effects.^37,59–63^ Our findings suggest that alterations in VCP function play a role in mutSOD1 toxicity, while VCP can modulate the degradation of insoluble mutSOD1 protein, linking two distinct genetic causes of ALS.

Our MS-based analysis identified VCP as a persisting protein in mutSOD1 MNs relative to isogenic control cultures. Proteins exhibiting slower turnover may have compromised function and contribute to neurodegeneration^64^ or persist to cope with additional workload. The beneficial effects of VCP expression in mutSOD1 MNs and a *C*. *elegans* model argue that the functional ability of VCP is impaired, or at the very least limited, by mutSOD1. Given that the function of VCP is tightly associated with its ability to interact with co-factors and substrate proteins,^42,65–67^ we examined the VCP interactome in mutSOD1 and control MNs. These experiments revealed that VCP exhibited a mutSOD1-dependent shift in the classes of proteins with which it interacts. This shift away from cytoskeletal proteins toward the enrichment for UPS-related proteins may reflect homeostatic alterations in these pathways, as MNs progressively accumulate disordered and misfolded SOD1. Critically, these pathways have been widely implicated in SOD1-ALS pathophysiology.^68–76^

### Limitations of the study

The underlying molecular mechanisms that trigger the slower degradation of VCP in mutSOD1 MNs remain unclear and will require further investigation in future studies. One possibility is that disordered or misfolded SOD1 triggers the slower turnover of VCP indirectly through common interaction partners or perhaps by increasing the workload of the ERAD pathway. Of note, mutSOD1 has been shown to induce ER stress in iPSC-derived MNs,^17^ while VCP has a well-defined role in ERAD.^56,77^ Intriguingly, a recent bioinformatics-based study that constructed a protein-protein interaction network associated with ALS highlighted SOD1 and VCP as two out of five proteins that are most vital for its sustainability, although there was no evidence for a direct interaction between the two proteins.^78^ Moreover, transcriptomic analysis flagged overlapping expression changes in modules between mutSOD1 and mutVCP MNs.^79^ At the same time our biochemical data in HEK293T cells demonstrate that overexpression of VCP can eliminate mutSOD1 protein accumulating in the detergent-insoluble fraction, while this effect can be reversed upon chemical inhibition of VCP activity. Additionally, the chemical inactivation of VCP in patient MNs further enhanced the accumulation of SOD1 in the detergent-insoluble fraction, demonstrating that VCP is associated with the clearance of misfolded proteins such as SOD1. Given that we did not identify SOD1 as a VCP-interacting protein, it is likely that these effects and the overall interplay between the two proteins are indirect.

Importantly, our work demonstrates that mutSOD1 does not cause a widespread imbalance to the proteome or severe disruptions to proteostasis mechanisms, but rather affects the turnover of a small and specific subset of proteins. The selective impairment in protein degradation likely reflects early cellular events in response to misfolded SOD1 protein. The enrichment for proteins associated with chaperone activity, folding of polypeptides in the ER, and cytoskeletal homeostasis are in line with previous reports of mutSOD1 toxicity in iPSC-derived neurons and during early disease stages in animal models.^17,18,79–81^ While MNs derived from iPSCs recapitulate the physiological expression of mutSOD1 in the context of each patient’s genetic background, they also have limitations associated with most *in vitro* model systems such as the lack of non-cell-autonomous contributions to pathophysiology. Most critically, iPSC-derived neurons resemble neurons of early postnatal developmental stages, at least based on their gene expression pattern,^82,83^ and thus do not recapitulate pathology associated with late disease stages. Nevertheless, these results substantiate the idea that gain-of-function effects of disordered SOD1 protein target selective neuronal pathways in the context of a model that does not require artificial overexpression. Our work links two distinct ALS-causal genes, highlights impaired protein degradation as an underlying disease mechanism, and raises the possibility that boosting the expression of VCP may be a promising therapeutic target in mutSOD1 patients with ALS.

## STAR★METHODS

### RESOURCE AVAILABILITY

#### Lead contact

Further information and requests should be directed to and will be fulfilled by the lead contact, Evangelos Kiskinis (evangelos.kiskinis@northwestern.edu).

#### Materials availability

All unique/stable reagents generated in this study are available from the lead contact with a completed Materials Transfer Agreement.

#### Data and code availability

- All data generated or analyzed during this study are included in the manuscript and supporting files. RAW MS data have also been deposited at MassIVE under the accession number MSV000092310 or at Proteome Exchange under the accession number PXD043406.
- The data are publicly available.
- This paper does not report original code.
- All data are available from the lead contact upon request.

### EXPERIMENTAL MODEL AND STUDY PARTICIPANT DETAILS

#### Directed differentiation of iPSCs to spinal motor neurons

Induced Pluripotent Stem Cells (iPSCs) were maintained and expanded as colonies on top of Corning Matrigel matrix (Corning 354277), fed with mTeSR1 (Stem Cell Technologies). For their differentiation into spinal motor neurons, we followed the standard 2D directed-differentiation protocol, previously reported in.^75^ Briefly, iPSCs were dissociated in single cells using Accutase, counted and plated in mTeSR and 10μM ROCK inhibitor (Y-27632, DNSK International) at a density of 1.2M/well of a 6-well plate. Next day (day 0) when the confluence into the well reached the 70–80%, the media was removed, cells were washed gently with PBS and fed daily with N2B27 media (50% DMEM:F12/50% Neurobasal media, supplemented with Non-Essential Amino Acids, Glutamax, N2, B27, all from Thermo Fischer Scientific) including 10μM SB431542 (DNSK International), 100nM LDN-193189 (DNSK International), 1μM Retinoic Acid (Sigma-Aldrich), 1μM of Smoothened-Agonist (SAG, DNSK International) in order to induce the neuralization in the culture. On day 6 the media was switched to N2B27 including 1μM Retinoic Acid, 1μM SAG, 5μM DAPT (DNSK International) and 4μM SU5402 (DNSK International), forcing the neuronal progenitors to exit the cell cycle. On day 14, the cells were dissociated using TryplE (Thermo Fischer Scientific) + DNaseI (Worthington) for 10 min at 37°C and plated on top of Matrigel at a density of 1M/well of a 6-well plate. The culture was maintained in NBM media (Neurobasal media, supplemented with Non-Essential Amino Acids, Glutamax, N2, B27) including the neurotrophic factors BDNF, CNTF, GDNF (all from R&D systems, at 10 ng/mL) and Ascorbic acid (0.2 μg/ml), until day 51 (every other day feeding). In order to inhibit the growth of proliferating progenitor cells, we add 10μM EdU to the media during the first week, which incorporates into DNA during S-phase and blocks cell proliferation.

#### *C*.*elegans* locomotor assays

The following strains of *C*.*elegans* were used in this study: N2 (Bristol), IW8 (Psnb-1G85R SOD1YFP), FX544 (cdc-48.1(tm544)), PP265 (N2; hhEx8[cdc-48.1cdc48.1GFP,ttx-3GFP], RK163 (Psnb-1G85R SOD1YFP; cdc-48.1(tm544)) and RK197 (Psnb-1G85R SOD1YFP; hhEx8[cdc-48.1cdc48.1GFP,ttx-3GFP). *C*. *elegans* locomotor behavior was tested in a swimming assay as described previously.^87^ For each assay, 15–20 worms were allowed to lay eggs for 4h. When animals reached the fourth larval stage (L4), individual animals were picked and suspended in a pool of M9 buffer (22 mM KH2PO4, 42 mM Na2HPO4, 86 mM NaCl) and their locomotor behavior was blindly recorded for 30 s on a video camera attached to a Zeiss Stemi SV11 dissecting scope. At least four independent experiments were performed for each assay. Three replicates containing 8–12 animals per group were tested. The video data of all worms tracked in movies were analyzed by NIH ImageJ software (http://www.phage.dk/plugins/wrmtrck.html)^88^ to obtain average speed (Length/time) as well as body bends per second (BBPS). Statistical analysis was performed using GraphPad Prism version 9.0.0 for MacOS. Values were tested for statistical significance using one-way ANOVA as well as unpaired t-tests between the groups of interest.

### METHOD DETAILS

#### Cell culture labeling (SILAC)

The Stable Isotope Labeling with Amino acids in Cell culture (SILAC) was performed as described previously with minor modifications.^89^ The MN in culture were fed for two weeks (days 16–30) with NBM-SILAC media (heavy, pulse), containing L-Lysine:2HCL (^13^C_6_, 99%; ^15^N_2_, 99%) and L-Arginine:HCL (^13^C_6_, 99%; ^15^N_4_, 99%), both from Cambridge Isotope Laboratories Inc. On day 30 the NBM-SILAC media was replaced with the regular NBM media (light, chase) containing non-labeled amino acids and the MN were collected at various time-points from day 30 up to day 51.

#### Sample preparation for mass spectrometry (MS)

The protein samples derived from either pulse-chased MN cultures at various time-points or from IP reactions (see Immunoprecipitation assays section) were quantified by BCA assay (Thermo Scientific 23227) and subjected to Trichloroacetic Acid (TCA) precipitation. In brief, the volume of the lysates was adjusted to 400μL with 100μM Tris-Cl pH 7.5 and the TCA was added at a final concentration 20% (v/v). The samples were vortex-ed, kept in an ice bucket at 4°C overnight and next day, they were span-down at 13,000 rpm, 4°C for 30min. The TCA was carefully removed, and the pellets were washed three times with 100% iced-cold methanol and dried at 95°C for 5min.

The precipitated samples were solubilized and denatured in 8M urea for 30min and processed with 0.2% ProteaseMAX (Promega V2072) for 2h. Subsequently, the samples were reduced with 5mM Tris(2-carboxyethyl) phosphine (TCEP) at room temperature (RT) and alkylated with 10mM iodoacetamide (IAA) while protected from light. Then, they were diluted with 50mM ammonium bicarbonate, quenched with 25mM TCEP and digested overnight at 37°C with 1μg sequencing-grade trypsin (Promega V5280). The digestion was terminated using 1% formic acid and the peptides were desalted using the HyperSep C18 Cartridges (Thermo Scientific 60108–302) and dried down by vacuum centrifugation. In the whole proteome analysis experiments the desalted peptides were fractionated using HyperSep Strong Cation Exchange (SCX) columns (Thermo Scientific 60108–420) based on the protocol of the manufacturer and the fractions were eluted in a wide range of ammonium acetate concentrations (20–2,000mM). The peptide fractions were again desalted with Pierce C18 spin columns (Thermo Scientific 89873) and dried down.

#### Liquid chromatography mass spectrometry (LC-MS/MS)

Dried peptide samples were resuspended in 20μL of Buffer A (94.875% H2O with 5% ACN and 0.125% FA), and 3μg sample was loaded via autosampler with UltiMate 3000 HPLC pump onto a vented Pepmap 100, 75 μm × 2 cm, nanoViper trap column coupled to a nanoViper analytical column (Thermo Fisher Scientific) with a stainless steel emitter tip assembled on the Nanospray Flex Ion Source with a spray voltage of 2,000 V. Buffer A contained 94.785% H2O with 5% ACN and 0.125% FA, and buffer B contained 99.875% ACN with 0.125% FA.

MS/MS data were obtained using Orbitrap Fusion with MS parameters including the following: ion transfer tube temp, 300°C; Easy-IC internal mass calibration; default charge state, 2; cycle time, 3 s; detector type set to Orbitrap; 60K resolution; wide quad isolation; mass range, normal; scan range, 300–1,500 m/z; maximum injection time, 50ms; AGC (automatic gain control) target, 200,000; microscans, 1; S-lens RF level, 60; without source fragmentation; and datatype, positive and centroid; MIPS set as on; included charge states, 2–6 (reject unassigned); dynamic exclusion enabled, with n = 1 for 30- and 45-s exclusion duration at 10 ppm for high and low; precursor selection decision, most intense, top 20; isolation window, 1.6; scan range, auto normal; first mass, 110; and collision energy, 30%. For collision-induced dissociation (CID): detector type, ion trap; OT resolution, 30K; IT scan rate, rapid; maximum injection time, 75 ms; AGC target, 10,000; Q, 0.25; and inject ions for all available parallelizable time. We performed 2- or 4-h analysis runs.

#### Tandem mass spectra analysis

The spectral files from all replicates were pooled for a single database search. Spectrum raw files were extracted into MS1 and MS2 files using in-house program RawXtractor or RawConverter (http://fields.scripps.edu/downloads.php).^90^ The tandem mass spectra were searched against UniProt human protein database (downloaded on 01–01-2014 or 03–25-2014; UniProt Consortium, 2015) and matched to sequences using the ProLuCID/SEQUEST algorithm (ProLuCID version 3.1^91,92^ with 50ppm peptide mass tolerance for precursor ions and 600 ppm for fragment ions. The search space included all fully and half-tryptic peptide candidates that fell within the indicated mass tolerance window with no miscleavage constraint, assembled, and filtered with DTASelect2 (version 2.1.3)^93,94^ through Integrated Proteomics Pipeline IP2 version 3, Integrated Proteomics Applications (http://www.integratedproteomics.com). In order to estimate peptide probabilities and false-discovery rates (FDR) accurately, we used a target/decoy database containing the reversed sequences of all the proteins appended to the target database.^95^ Each protein identified was required to have a minimum of one peptide of minimal length of six amino acid residues; however, this peptide had to be an excellent match with an FDR = 0.001 and at least one excellent peptide match. After the peptide/spectrum matches were filtered, we estimated that the protein FDRs were <1% for each dataset. Resulting protein lists include subset proteins to allow for consideration of all possible protein forms implicated by a given peptide identified from the complex protein mixtures.

Labeling efficiency was determined by MS analysis of day 30 when the heavy cultures were lysed.^96^ We used the Prolucid database search engine with a combined heavy/light mouse protein database, filtered our results with DTASelect (protein FDR<1%, peptide FDR<0.3%) and used Census to determine the heavy/light ratio for each peptide from the area under each curve. We binned peptides based on common ratios and graphed their distribution and determined the average enrichment. In our calculations a peptide enrichment ratio of 90 is equal to ~90% labeled.^97^

We downloaded the individual Census output file and extracted the quantitative measure of each heavy peptide abundance (i.e., area under the reconstructed chromatogram). For each protein we averaged the quantitative measure of each peptide (including singletons). We only considered proteins that were quantified on day 30 and at least one additional time-point. We then calculated the ratio of the heavy protein on the time-point of interest relative to day 30. We only considered proteins in which this ratio was lower than 1.

To analyze the LC-MS/MS interactome data derived from the immunoprecipitation of VCP we first calculated the ratio of the spectra counts for each protein over the spectra counts of VCP. We subsequently classified proteins into ones that co-precipitated exclusively with VCP and ones that co-precipitated with both VCP and IgG. For proteins that co-precipitated with both VCP and IgG, we calculated the spec count ratio of VCP over IgG and considered as genuine VCP interactors only ones where this ratio was >3. We conducted this process for each one of the three independent immunoprecipitation assays and for each one of the two genotypes separately. These VCP-interacting proteins were subsequently used for comparing mutSOD1 and isogenic control MNs.

To compare interactors between mutSOD1 and isogenic control MNs, we only considered proteins that we identified as VCP-interactors by the criteria described above, in all three independent immunoprecipitation assays. This analysis yielded 153 VCP-interacting proteins that were exclusively identified in mutSOD1 MNs, and 157 VCP-interacting proteins that were exclusively identified in isogenic control MNs. Additionally, we identified 399 VCP-interacting proteins that were common between mutSOD1 and isogenic control MNs. To identify enriched interactors within one of the two genotypes from the group of common interactors we performed multiple unpaired t tests and considered a p value of <0.05 as significant, yielding 10 VCP-interacting proteins enriched in mutSOD1 MNs, and 19 VCP-interacting proteins enriched in isogenic control MNs.

#### ELISA

Disordered SOD1 was quantified using a specific ELISA (misELISA) described previously.^36^ The protocol has been validated extensively in patient derived fibroblasts and MNs.^34,35^ For the preparation of samples, MNs were washed with pre-warmed PBS-Iodoacetamide (IAM) 40mM, an alkylating agent to block free thiol groups in the cysteine residues of disordered SOD1 and prevent refolding. Cells were dissociated in 0.025% (w/v) trypsin/PBS-IAM 40mM for 5min, collected and centrifuged at 500×g for 5min. The cell pellet was resuspended in 1mL PBS-IAM and transferred to a fresh 1.5mL tube. The cells were then re-centrifuged at 500×g for 5min, the supernatant was removed, and the pellet was stored at −80°C until further analysis.^34^

#### Biochemical fractionation of iPSC-derived MNs for WB analysis

The protocol has been adapted by^35^ with minor modifications. Briefly, cells (~1M) were harvested in 130μL NP-40 lysis buffer (PBS, 0.5% NP-40, complete protease inhibitors w/o EDTA, Calbiochem), sonicated 3 × 3s, 35V output (QSonica, LLC) and centrifuged at 20,000×g for 30min. The supernatant designated as the “soluble fraction” was retained and the pellet was further washed twice with NP-40 lysis buffer and thereafter designated as the “insoluble fraction”. After each wash the pellet was centrifuged at 20,000×g for 30min. Following the second wash the pellet was re-suspended in 15μL NP-40 lysis buffer and briefly sonicated for 3s to facilitate homogenization. Both soluble (20–30μg) and insoluble (total volume) fractions were loaded for SDS-PAGE and WB analysis.

#### Isolation of poly-ubiquitinated proteins using TUBE magnetic beads

For the isolation of Poly-Ubiquitinated proteins from MN lysates we performed pull down assays using TUBE magnetic beads (Tandem Ubiquitin Binding Entities, Life Sensors, UM402M) according to the protocol described by the manufacturer. In brief, MNs (~3M) were collected in TUBE lysis buffer (50mM Tris-HCl, pH 7.5, 0.15M NaCl, 1mM EDTA, 1% NP-40, 10% glycerol, protease inhibitors), sonicated 3 × 3s, 35V output (QSonica, LLC) and clarified by high-speed centrifugation (~14,000×g) for 10 min at 4°C. An “input” sample was removed for analysis by western blotting and approximately 500μg of protein lysate (determined by BCA quantification) was subjected to pull-down. Cell lysate was mixed with equilibrated Magnetic-TUBE beads and incubated for 3h at 4°C. The TUBE beads were isolated using a magnetic stand, washed three times with 1mL TBS-Tween 0.1% (TBST) and poly-ubiquitinated proteins were eluted in Laemmli SDS loading buffer including β-Mercaptoethanol. The samples were boiled for 10 min at 95°C, spun down (13,000×g for 5min) and loaded on poly-acrylamide gels for SDS-PAGE/WB.

#### DSP cross-linking

We used the DSP cross-linker (Thermo, 22585) following the instructions of the manufacturer to crosslink MN cultures and analyze the interactome of VCP. Briefly, the DSP powder was dissolved in DMSO at a stock concentration of 10mM and then diluted 1:10 (1mM working concentration) in PBS. MN cultures were washed once with PBS and incubated with the DSP solution for 30 min at RT. Subsequently, the DSP solution was removed and the MNs were incubated with the Stop Solution (10mM Tris pH 7.5) for 15 min at RT. The Stop Solution was then removed, and the cells were lysed in IP lysis buffer (HEPES pH 7.6 10mM, NaCl 100mM, Sodium Deoxycholate 1%, SDS 0.1%, Triton X-100 1%, Glycerol 10%, protease and phosphatase inhibitors) w/o DTT for immunoprecipitation.

#### Immunoprecipitation assays

For the immunoprecipitation assays we used the Protein A Magnetic Dynabeads (Life Technologies, 10001D) following the instructions of the manufacturer with minor modifications. First, we added 50μL of Dynabeads (per reaction) to a 1.5mL tube and placed to a magnetic stand to remove the beads from the solution. The antibody (5μg in 200μL PBS-Tween 0.02%) was then mixed with the Dynabeads and incubated under rotation for 30 min at RT. The tube was again placed on the magnet to remove the supernatant and beads were washed with 200μL PBS-Tween 0.02%. The beads were incubated with the lysate (500–1000μg) for 1h at RT. The supernatant (unbound fraction) was isolated for further analysis and the beads were washed 3 times with 200μL PBS-Tween 0.02%. Precipitated proteins were eluted in Laemmli sample buffer including β-mercaptoethanol. Samples were boiled for 10 min at 95°C, span down at 13,000×g for 5min and analyzed using SDS-PAGE/WB. Immunoprecipitates used for MS analysis, were eluted in a Laemmli sample buffer including β-mercaptoethanol but free of bromophenol blue and the samples were subjected to TCA precipitation (See section Sample preparation for Mass Spectrometry).

#### Western blot analysis

Whole cell lysate extracts in RIPA buffer (Tris pH 7.4 50mM, NaCl 150mM, Sodium Deoxycholate 0.5%, Triton X-100 1%, SDS 0.2%, EDTA 1mM, protease and phosphatase inhibitors) or cellular fractions (soluble and insoluble) in NP-40 lysis buffers (including protease inhibitors) were quantified for their protein concentration using the Pierce BCA Protein Assay Kit (Cat No 23227). A total of 20μg of protein samples were mixed with Laemmli SDS loading buffer including β-Mercaptoethanol, boiled for 10 min at 95°C and loaded onto 4–20% Mini-PROTEAN Stain-Free Gels (BioRad, Cat No 4568094) for SDS-PAGE under constant voltage (100V) for 1.5h. The gels were then activated under UV-light for 5min using the ChemiDoc MP imaging system from BioRad and the proteins were transferred to nitrocellulose membrane (BioRad, Cat No 1620115, 0.45μM) under constant voltage (100V) for 1h at 4°C. The transferred proteins onto the membrane representing the total protein load, were visualized with the ChemiDoc MP imaging system, and used for normalizing the abundance of target proteins. The membrane was blocked with 5% non-fat milk in TBS-Tween 0.1% at 37°C for 30min and probed overnight at 4°C with specific antibodies (diluted in 2.5% non-fat milk in TBS-Tween 0.1%) against antigens of interest. Next day, the membrane was washed three times x 10min with TBS-Tween 0.1% under agitation and probed with HRP-conjugated antibodies (1.5 h at RT under agitation), that bind to the IgG portion of primary antibodies. After membrane washes, the bands of interest were detected by Enhanced Chemiluminescence using the respective kits from Thermo Fisher Scientific (SuperSignal West Pico PLUS Chemiluminescent Substrate or SuperSignal West Femto PLUS Chemiluminescent Substrate).

#### Immunocytochemistry

The iPSC-derived MNs were plated on day 14, on top of 1mm glass coverslips (Fisher scientific) coated with Matrigel (37°C, overnight) at a density of 80,000 cells/coverslip. Cells were washed once with PBS and fixed with 4% PFA in PBS for 20 min at RT. Following fixation cells were washed three times with PBS and permeabilized with PBS-Triton X-100 0.2% for 45 min at RT. To block the non-specific binding of primary antibody to unrelated epitopes, cells were treated with 10% Normal Donkey Serum (Jackson ImmunoResearch) in PBS-Triton X-100 0.1%, for 1h at RT and then incubated with primary antibody, diluted in 2% Normal Donkey Serum in PBS-Triton X-100 0.1%, overnight at 4°C. Next day, coverslips were washed three times with PBS and incubated with the fluorophore-conjugated secondary antibody (Alexa Fluor 488, or Alexa Fluor 647 or Alexa Fluor 555, all from Invitrogen) at a dilution 1:1,000 in 2% Normal Donkey Serum in PBS-Triton X-100 0.1%, for 2h at RT protected from light. Cells were washed once with PBS, incubated with DAPI 1:1,000 in PBS for 10 min at RT, washed again three times with PBS and coverslips were mounted on glass slides using Fluoromount-G (Southern Biotech).

#### Plasmid construction

The SOD1WT-MYC and SOD1A4V-MYC plasmids, used for the HEK293T cell transfection, were generated as follows: the human SOD1WT and SOD1A4V cDNAs included in the pDONR221 plasmids (gift from Kevin Eggan, Harvard University) were amplified by PCR using the primers SOD1WTFOR, SOD1A4VFOR, SOD1REV (see table below). The SOD1WTFOR and SOD1A4VFOR primers include the recognition site of the restriction endonuclease NheI and the SOD1REV primer includes the recognition site of the restriction endonuclease XhoI. The amplified products upon digestion with the aforementioned enzymes and cleaning (Wizard SV Gel and PCR Clean-Up System Protocol, Promega) were subcloned into the pLenti-*c*-Myc-DDK plasmid (gift from Jeffrey Rothstein,^98^ Johns Hopkins University). For the ligation reaction, the Quick Ligation Kit (NEB, Cat No M2200S) was used and the NEB 5-alpha Competent E. coli bacteria (Cat No C2987H), were then transformed with the ligation product. The pLV[Exp]-EF1A > hVCP [NM_007126.3](ns):T2A:TurboRFP (VB180718–1097hpn) and pLV[Exp]-EF1A>TurboRFP (VB900088–2446mnh) plasmids (as well as the respective viruses) were purchased by VectorBuilder.

**Table:** 

Primer name	Direction	Sequence

SOD1WTFOR	Forward	TAAGCAGCTAGCATGGCGACGAAGGCCGTGT
SOD1A4VFOR	Forward	TAAGCAGCTAGCATGGCGACGAAGGTCGTGT
SOD1REV	Reverse	TGCTTACTCGAGGTATTGGGCGATCCCAATTACACC

#### HEK293T transient transfection

HEK293T cells were plated in 12-w plates at a density of 100k/well and left to grow until they reach a 40% confluence. The transfection was performed using the HilyMax reagent and applying the respective protocol provided by the manufacturer (Dojindo Molecular Technologies, Inc., Cat. No H357). 24 or 48h upon transfection, when the culture reaches an 80% confluence, the cells were treated with either DMSO or NMS873 inhibitor at 10μM for 8h. The cells were then lysed and fractionated into soluble and insoluble fractions for WB analysis.

#### SOD1 gene editing in HUES3 ESCs

The generation of the HUES3-SOD1^+/A4V^ and isogenic control stem cell lines using Zing Finger Nucleases and their characterization has been previously described.^40^

#### C.elegans biochemical fractionation

The biochemical fractionation of worms was performed following a protocol described previously.^47^ Briefly, the worms were washed with lysis buffer (50mM KCl, 10 mM Tris-Cl, pH 8.3, 2.5mM MgCl2, 0.45% Tween 20, 0.45% NP-40, and 0.01% gelatin, supplemented with protease and phosphatase inhibitors). The samples were centrifuged once, resuspended in 200μL lysis buffer, and sonicated twice: for each round of sonication the standard pulse protocol was applied (3 × 3sec with 3sec interval, at 35 Ampl. The samples were then centrifuged at 99,000×g, at 4°C for 30min and the supernatant that contains the soluble proteins, was saved for SDS-PAGE and WB analysis. The pellet was resuspended in lysis buffer and centrifuged again at 99,000×g, under the same conditions. The supernatant was removed and the remaining pellet that contains the insoluble proteins was subjected to SDS-PAGE and WB analysis.

#### Induced motor neuron (iMN) generation and survival assay

NGN2, ISL1, LHX3 (NIL)-driven iMNs were made as previously described.^99^ Briefly, iPSCs were seeded in Matrigel coated 24-well plates at 80–120k cells/well in mTeSR1 supplemented with 10μM ROCK inhibitor (Ri; Selleck). The next day, cells were infected with a lentivirus encoding NIL, and a lentivirus encoding rtTA3. Induction media (DMEM/F12, GlutaMAX, NEAA, N2 supplement, 0.2μM compound E, 10 ng/ml BDNF, 10 ng/ml NT3, 10 ng/ml GDNF and 10 ng/ml CNTF) with 10μM Ri and 1 μg/ml doxycycline was added to the infected cells to drive the NIL over-expression and the puromycin resistance after a 1:2 replating. Puromycin at 0.5 μg/ml was added to the induction media two days later for 24h to remove non-infected iPSCs. On the next day, converting iMNs were replated again on monolayers of primary rodent glia to support maturation of neurons, in N3 media with BrdU at 40μM to eliminate any dividing cells. Doxycycline was maintained in the medium for 10 days. Medium was refreshed every other day.

SynGFP+ iMNs formed between days 13–16 after transduction of iMN factors. LV-VCP-RFP/LV-VCP infection was done on day 14. A complete media change was made on day 15. The iMN survival assay was initiated on day 17. Neurotrophic factor withdrawal (BDNF, GDNF, FGF, and CNTF) were removed from the culture medium on day 17. Starting at Day 17, longitudinal tracking of iMNs was performed using Molecular Devices ImageExpress once every other day for up to 20 days. Tracking of neuronal survival was performed using SVcell 3.0 (DRVision Technologies) or ImageJ. Neurons were scored as dead when their soma was no longer detectable by GFP fluorescence. The media was changed every other day.

#### Immunohistochemistry

The Johns Hopkins ALS Postmortem Core provided formalin-fixed paraffin-embedded sections of human postmortem brain tissue from the motor cortex and the occipital cortex of each decedent. Tissue slides were submerged sequentially in xylene (Sigma Aldrich) three times for 10 min each, 100% ethanol (Decon Laboratories) three times for 5 min each, 95% ethanol three times for 3 min each, 75% ethanol for 2 min, and 50% ethanol for 2 min. Slides were rinsed gently in DI water five times with light shaking and dried. Slides were then placed in decloaker solution (135mL DI water, 15mL Antigen Decloaker 10X, BioCare Medical) and incubated in decloaking chamber (Instapot) for 10min. Slides were then cooled and rinsed gently in DI water five times with light shaking and dried. Next, a Pap Pen (Vector Laboratories) was used to outline the tissue section on each slide. Slides were then treated with 200μL of 1% BSA in 1X PBS for 20 min at RT. Slides were next rinsed gently in DI water five times with light shaking and dried. Primary antibodies were diluted in 1% BSA in 1X PBS: SOD1 (rabbit, 1:100, Abcam), VCP (mouse, 1:100, Genetex), and MAP2 (chicken, 1:100, Abcam). Slides were treated with primary antibody solution and incubated overnight at 4°C. The following day slides were next rinsed gently in DI water five times with light shaking and dried. Secondary antibodies (Alexa Fluor 488 goat anti-mouse, Alexa Fluor 594 donkey anti-rabbit, and Alexa Fluor 647 donkey anti-chicken 647) were diluted in 1X PBS and conjugated to primary antibodies for 1h at RT. Slides were next rinsed gently in DI water five times with light shaking and dried. DAPI was diluted 1:200 in 1X PBS and added onto the slides for 1h at RT. Slides were next rinsed gently in DI water five times with light shaking and dried. Next, Sudan Black (Millipore Sigma, prepared by adding 105mg Sudan Black to 35mL 70% ethanol and filtering through Whatman paper for 2 min) was added on the slides for 40 s at RT. Slides were then rinsed gently in DI water fifteen times with light shaking and dried. Slides were then mounted on Fluoromount-G (Southern Biotech) with a No.1 cover glass (Fisher Scientific, 60 × 24 mm) and dried overnight at RT. Nail polish was used to seal the edges the following day.

Images used for quantification were acquired at matched exposure times and identical laser settings between comparative conditions. Image acquisition was performed on a Nikon W1 dual camera spinning disk confocal microscope (Northwestern University Center for Advanced Microscopy) through z-stacking at 0.3μm intervals. The following exposure times were used to image in each channel: MAP2 (647) – 2s; SOD1 (555) – 4s; VCP (488) – 3s; DAPI (405) – 3s. Individual planes were combined into a 3-D reconstruction using IMARIS software (ver. 9.9.0, Northwestern University Center for Advanced Microscopy). Regions of interest, neurons, were made by generating surfaces of high MAP2 signal intensity. The surface grain size parameter was set to 0.700μm. To maximize the area of each neuron in the surface generated, the absolute threshold and number of voxels per image was varied per image. Within these surfaces, spots of high VCP or SOD1 signal above a certain threshold were generated. The diameter of VCP and SOD1 spots was set to 0.500μm and 0.600μm, respectively. The “quality” of these spots was used to threshold them, which is based on the signal to noise ratio. In JHU14, the quality of VCP and SOD1 spots was set to 1,000 and 170, respectively. In JHU74, the quality of VCP and SOD1 spots was set to 200 and 50, respectively. Finally, the mean intensities of these spots were outputted. These mean intensities were averaged within each neuron, and those data were graphed.

#### 20S proteasome activity assay

For the quantification of the 20S proteasome activity in MN lysates, we used a 96-well microplate-based assay kit provided by Chemicon (Cat. No. APT280) and followed the manufacturer’s protocol. In brief, lysates from either SOD1^+/A4V^ or SOD1^+/+^ MN on days 30 and 35, were isolated in RIPA lysis buffer without proteasome inhibitors, so we could measure proteasome-dependent degradation. We used the labeled substrate LLVY-AMC which upon cleavage releases the fluorophore AMC (7-Amino-4-methylcoumarin) that can be quantified fluorometrically (389/460nm excitation/emission). An AMC standard curve was also generated for the calculation of fluorescence derived from the experimental samples, while a negative control (test sample without substrate) was used in parallel. All the samples were incubated within the 96-well microplate for 2h at 37°C before measuring the fluorescence signal.

#### Lysosomal activity assay

For the lysosomal activity assay, SOD1^+/A4V^ or SOD1^+/+^ MN were plated on a 96w plate at a density of 60,000 cells/well and were incubated with Dextran Blue (1 mg/ml working concentration) for 24h. Next day, the specific inhibitor Bafilomycin A1, which disrupts lysosomal pH and reduces the lysosomal activity of Glucocerebrosidase (GCase), was added to the culture at 200nM working concentration, while control wells were treated with the vehicle DMSO. One hour later the Dextran Blue was washed out and the cells were pulsed with the fluorescent substrate of the enzyme GCase, PFB-FDGlu (P11947 Invitrogen) at 100 μg/ml working concentration for 1h. Following incubation, the cells were washed once with media and phenol-free NBM was added to the wells. The fluorescence of PFB-FDGlu (485/535nm excitation/emission) was measured with microplate reader (Spectramax Gemini Microplate Reader, Molecular Devices) every 0.5h for up to 3h and normalized to the lysosomal mass (Dextran Blue, ex = 400, em = 430). In order to evaluate the selective lysosomal activity for each type of MN, the fluorescence derived from control wells treated with DMSO was subtracted from the respective wells treated with Bafilomycin A1.

#### Gene ontology analysis

The Gene Ontology (GO) analysis was performed using the PANTHER database (versions 16.0 and 17.0)^84^. For the visualization of persisting proteins and their functional association the STRING database^100^ was used. In addition, the WebGestalt online tool for the GO enrichment analysis was used.^86^ For the generation of Venn diagrams the online tool Meta-Chart (https://www.meta-chart.com/) was used.

### QUANTIFICATION AND STATISTICAL ANALYSIS

#### Statistical analysis

The quantification and statistical analysis for all the experimental assays were performed using the GraphPad Prism 9.1.2.226 and Fiji (former ImageJ) software (https://imagej.nih.gov/ij/).

## Supplementary Material

1

2

3

4

5

## Figures and Tables

**KEY RESOURCES TABLE:** 

REAGENT or RESOURCE	SOURCE	IDENTIFIER

Antibodies		

Anti-VCP mouse primary antibody	Genetex	GTX101089; RRID:AB_1952544
Anti-SOD1 rabbit primary antibody	Abcam	ab51254; RRID:AB_882757
Anti-MAP2 chicken primary antibody	Abcam	ab5392; RRID:AB_2138153
Anti-β-Tubulin III rabbit primary antibody	Millipore-Sigma	T2200–200UL; RRID:AB_262133
Anti-Choline Acetyltransferase goat primary antibody	Millipore-Sigma	AB144P-200UL; RRID:AB_2079751
Anti-ISL1/2 mouse primary antibody	DSHB (University of Iowa)	39.4D5; RRID:AB_2314683
ms SODI 134.2 (57–72)	Umeå University	Zetterstrom et al., 2011
rb SODI (24–39)	Umeå University	Zetterstrom et al., 2011
VCP	Abcam	ab109240; RRID:AB_10862588
Ub (P4D1)	Santa-Cruz Biotechnology	sc-8017; RRID:AB_628423
p62/SQSTM1	Proteintech	18420–1-AP; RRID:AB_10694431
HSPB1	Proteintech	18284–1-AP; RRID:AB_2295540
Normal Rabbit IgG	Cell Signaling	2729S; RRID:AB_1031062
Myc-Tag (9B11)	Cell Signaling	2276S; RRID:AB_331783
Alexa Fluor 488 goat anti-mouse secondary antibody	Jackson Immuno Research (Fisher Scientific)	A11001; RRID:AB_2534069
Alexa Fluor 594 donkey anti-rabbit secondary antibody	Jackson Immuno Research (Fisher Scientific)	A21207; RRID:AB_141637
Alexa Fluor 647 donkey anti-chicken secondary antibody	Jackson Immuno Research (Fisher Scientific)	703–606-155; RRID:AB_2340380
Donkey anti-Mouse IgG (H + L) Highly Cross-Adsorbed Secondary Antibody, Alexa Fluor™ 488	Invitrogen	A21202; RRID:AB_141607
Donkey anti-Goat IgG (H + L) Cross-Adsorbed Secondary Antibody, Alexa Fluor™ 488	Invitrogen	A11055; RRID:AB_2534102
Donkey anti-Rabbit IgG (H + L) Highly Cross-Adsorbed Secondary Antibody, Alexa Fluor™ 647	Invitrogen	A31573; RRID:AB_2536183
DAPI	Invitrogen	H21492

Cell Culture Reagents		

mTeSR	Stem Cell Technologies	5820
N-2 Supplement (100X)	Thermo Fisher Scientific	17502001
B-27 Supplement (50X), serum free	Thermo Fisher Scientific	17504001
GlutaMAX Supplement	Thermo Fisher Scientific	35050061
MEM Non-Essential Amino Acids Solution (100X)	Thermo Fisher Scientific	11140050
DMEM/F-12	Thermo Fisher Scientific	11320082
Neurobasal Medium	Thermo Fisher Scientific	21103049
EdU	Thermo Fisher Scientific	A10044
Trypsin-EDTA (0.25%), phenol red	Thermo Fisher Scientific	25200072
TrypLE™ Express Enzyme (1X), no phenol red	Thermo Fisher Scientific	12604013
1X PBS	Corning	MT21040CV
Ultrapure water with 0.1% Gelatin	Millipore	ES006B
Accutase	Innovative Cell Technologies	AT 104–500
SB431542	DNSK International	DNSK-KI-12
SAG	DNSK International	DNSK-SMO-1
SU5402	DNSK International	DNSK-KI-11
Y27632. 2HCl	DNSK International	DNSK-KI-15–02
Retinoic Acid	Millipore-Sigma	R2625–50MG
L-Ascorbic Acid	Millipore-Sigma	A4403–100MG
DMSO (Tissue Culture)	Millipore-Sigma	D2650–100ML
DMSO (Sterile, filtered)	Tocris (Bio-Techne)	3176
LDN 193189 dihydrochloride	Bio-Techne	6053/10
DAPT	Bio-Techne	2634/10
Recombinant Human BDNF Protein, CF	Bio-Techne	248-BDB-050/CF
Recombinant Human CNTF Protein, CF	Bio-Techne	257-NT-050/CF
Recombinant Human GDNF Protein, CF	Bio-Techne	212-GD-050/CF
Matrigel	Corning	354277
SILAC Neurobasal [-] L-Arginine HCI, [-] L-Glutamine, [-] L-Lysine HCI	Gibco	ME100240L2
L-LYSINE:2HCL (13C6, 99%; 15N2, 99%)	Cambridge Isotope Laboratories, Inc.	CNLM-291-H-PK
L-ARGININE:HCL (13C6, 99%; 15N4, 99%)	Cambridge Isotope Laboratories, Inc.	CNLM-539-H-PK

Biological samples		

Patient 1 (JHU74), postmortem ALS brain tissue from motor and occipital cortex	Johns Hopkins University	SOD1^+/A4V^, Male,47 (age of death)
Patient 2 (JHU14), postmortem ALS brain tissue from motor and occipital cortex	Johns Hopkins University	SOD1^+/A4V^, Male,49 (age of death)

Chemicals, buffers, peptides		

Precision Plus Protein™ All Blue Prestained Protein Standards	BioRad	1610373
Restore Western Blot Stripping Buffer	Thermo Scientific	21059
DSP	Thermo	22586
Iodoacetamide	GE Healthcare	RPN6302
Trichloroacetic acid solution	Sigma	T0699
Igepal (NP-40)	Sigma	I8896
Triton X-100	Sigma	T8787
10% Tween 20 Solution	BioRad	1610781
4x Laemmli Sample buffer	BioRad	1610747
2-Mercaptoethanol	Sigma	M3148
Dextran Blue (DB)	Life Technologies	D1976
PFB-FDGlu (5-(Pentafluorobenzoylamino)Fluorescein Di-β-D-Glucopyranoside)	Thermo Scientific	P11947
10xTris/Glycine/SDS Buffer	BioRad	1610732
10x Tris/Glycine Buffer	BioRad	1610734
Protease Inhibitor Coctail Set III, EDTA-Free	EMD Millipore	539134–1ML
Phosphatase Inhibitor Coctail II	Abcam	ab201113
InSolution™ MG-132	EMD Millipore	474791
VCP Inhibitor III, NMS873	EMD Millipore	531088
Bafilomycin A1	ChemCruz	sc-201550
Xylene	Millipore-Sigma	XX0060–4
Ethanol	Decon Laboratories (Fisher Scientific)	4355223
Decloaker Solution 10X	BioCare Medical (Fisher Scientific)	CB910M
Pap Pen	Vector Laboratories (Fisher Scientific)	H-4000
BSA Fraction V	Millipore-Sigma	2930–100GM
Sudan Black	Millipore-Sigma	199664–25G
Fluoromount-G	Southern Biotech (Fisher Scientific)	0100–01

Cover Glass	VWR	48393106

Critical commercial assays/kits		

20S Proteasome Activity Assay Kit	EMD Millipore/Chemicon	APT280
TUBE Magnetic beads	Life Sensors	UM402M
Dynabeads® Protein A	Life Technologies	10002D
QIAGEN Plasmid Plus Midi Sample Kit	QIAGEN	12943
Wizard® SV Gel and PCR Clean-Up System	Promega	A9282
Quick Ligation™ Kit	NEB	M2200S
Pierce™ BCA Protein Assay Kit	Thermo Scientific	23227
SuperSignal™ West Pico PLUS Chemiluminescent Substrate	Thermo Scientific	34577
SuperSignal™ West Femto Maximum Sensitivity Substrate	Thermo Scientific	34095

Deposited data		

Raw Mass Spectrometry Data Files	This paper	MSV000092310

Experimental models: Cell lines		

iPSC/39b	Kiskinis et al.^17^	SOD1^+/A4V^, Female,43 (age of ALS onset)
iPSC/39b 2.5-Correcetd	Kiskinis et al.^17^	N/A
Stem Cells/HUES3 Hb9::GFP	Thams et al.^40^	CVCL_X724
Stem Cells/HUES3 Hb9::GFP SOD1^+/A4V^	Thams et al.^40^	N/A
HEK293T	ATCC	CVCL_0063

Experimental models: Organisms/strains		

*C.elegans*: WT	Oxford University	N2 (Bristol)
*C.elegans*: mutSODI (G85R)	Wang et al.^49^	IW8
*C.elegans*: cdc48.1 ko	The National BioResource Project:*C.elegans*	FX544
*C.elegans*: cdc48.1 o/e	Janiesch et al., 2007^84^	PP265
*C.elegans*: mutSOD1xcdc48.1 ko	This paper	RK163
*C.elegans*: mutSOD1xcdc48.1 o/e	This paper	RK197

Bacterial and Virus strains		

NEB® 5-alpha Competent E. coli	NEB	C2987H
pLV[Exp]-EF1A > hVCP[NM_007126.3](ns): T2A:TurboRFP	VectorBuilder	VB180718–1097hpn
pLV[Exp]-EF1A>TurboRFP	VectorBuilder	VB900088–2446mnh

Software and algorithms		

Prism 9.1.2.226	GraphPad Software	https://www.graphpad.com/
Fiji (former ImageJ)	NIH	https://imagej.nih.gov/ij/
PANTHER database (versions 16.0 and 17.0)	Mi et al.^85^	https://www.pantherdb.org/
WebGestalt	Zhang et al.^86^	https://www.webgestalt.org/
Meta-Chart	Online	https://www.meta-chart.com/
Adobe Illustrator 26.0.2	Adobe Creative Cloud	N/A
Adobe Photoshop 24.7	Adobe Creative Cloud	N/A

## Highlights

- A selective group of proteins degrade slower in mutSOD1 compared to isogenic control MNs
- VCP plays a key role in protein clearance mechanisms and persists in mutSOD1 MNs
- VCP exhibits an altered interactome and can modulate the degradation of mutSOD1
- VCP overexpression rescues mutSOD1 toxicity *in vitro* and *in vivo*
