# Supplementary material for: Analysis of proteome-wide degradation dynamics in ALS SOD1 iPSC-derived patient neurons reveals disrupted VCP homeostasis
Source: Cell Rep. Author manuscript; Available in PMC 2024 Jan 12. (PMC10785776; doi:10.1016/j.celrep.2023.113160)
Supplement: 1 [file NIHMS1941862-supplement-1.pdf]

Cell Reports, Volume *42*

Supplemental information

Analysis of proteome-wide degradation dynamics in ALS SOD1 iPSC-derived patient neurons reveals disrupted VCP homeostasis

Konstantinos Tsioras, Kevin C. Smith, Seby L. Edassery, Mehraveh Garjani, Yichen Li, Chloe Williams, Elizabeth D. McKenna, Wenxuan Guo, Anika P. Wilen, Timothy J. Hark, Stefan L. Marklund, Lyle W. Ostrow, Jonathan D. Gilthorpe, Justin K. Ichida, Robert G. Kalb, Jeffrey N. Savas, and Evangelos Kiskinis

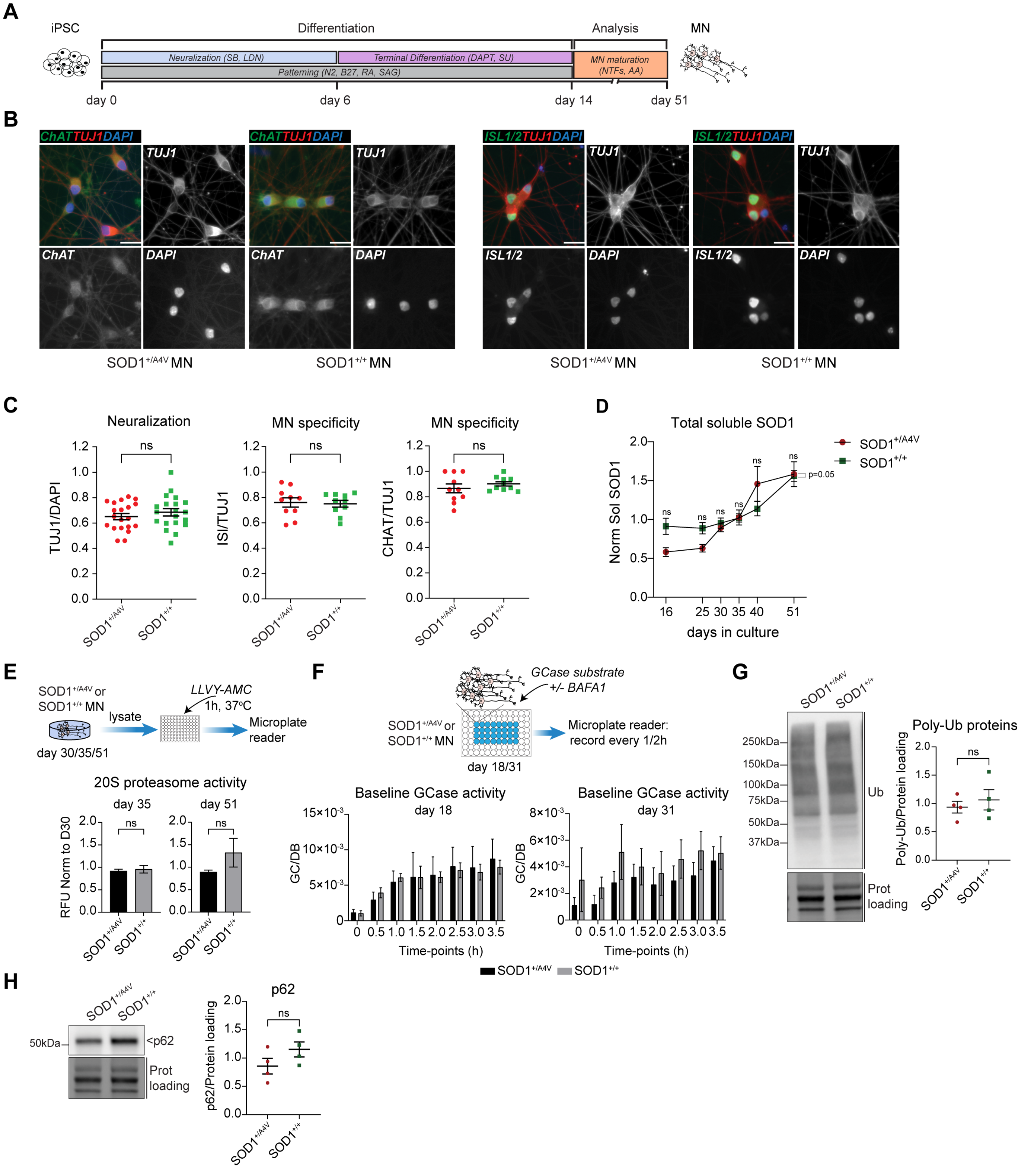

Supplementary Figure S1. MN differentiation efficiency, quantification of SOD1 protein and assessment of degradation mechanisms activity. Related to Figure 1.

(A) Schematic of the iPSC-based 14-day MN differentiation protocol.

(B) Representative images of iPSC-differentiated MNs expressing ISL1/2, ChAT and TUJ1 on day 25 (Scale bar = 20μM).

(C) Assessment of efficiency of neuralization (TUJ1/DAPI on day 16) and motor neuron specificity (ISL/TUJ1 and ChAT/TUJ1 on day 31) of MN cultures. Unpaired t-test, for TUJ1/DAPI n=20 independent fields, p=0.364; for ISL/TUJ1 n=10 independent fields, p=0.826; for ChAT/TUJ1 n=10 independent fields, p=0.375; ns=not significant. Each field contained more than 20 cells on average.

(D) Quantification of total soluble SOD1 by WB analysis in MN cultures. Two-way ANOVA across time and genotype: p=0.0497 for days, ns for genotype; Sidak's multiple comparisons test per time-point, d16 p=0.1958, d25 p=0.4602, d30 p=0.9993, d35 p>0.9999, d40 p=0.1607, d51 p>0.9999, n=6 independent biological replicates; ns=not significant.

(E) Experimental schematic (top) and quantification of the 20S proteasome activity in MNs under baseline conditions over-time (bottom). The relative fluorescence unit (RFU) values are normalized to day 30 for each corresponding genotype. Unpaired t-test, n=3 independent differentiations; for day 35 p=0.698, for day 51, p=0.249.

(F) Experimental schematic (top) and quantification of the lysosomal glucocerebrosidase (GCase) activity in MNs under baseline conditions in two time-points (day 18 and day 31). The columns represent the delta between DMSO and BAFA1-induced activity. The GCase activity is expressed relative to the volume of lysosomes (DB, Dextran Blue). Two-way ANOVA across time and genotype p=0.983, Sidak multiple comparisons test per time-point p>=0.99 for all the time-points examined, n=3 independent differentiations.

(G) Levels of poly-Ubiquitinated proteins under baseline conditions in mutSOD1 and isogenic control MNs. Unpaired t-test, n=4 independent differentiations, p=0.552; ns=not significant.

(H) SQSTM1/p62 levels under baseline conditions in mutSOD1 and isogenic control MNs. Unpaired t-test, n=4 independent differentiations, p=0.173; ns=not significant.

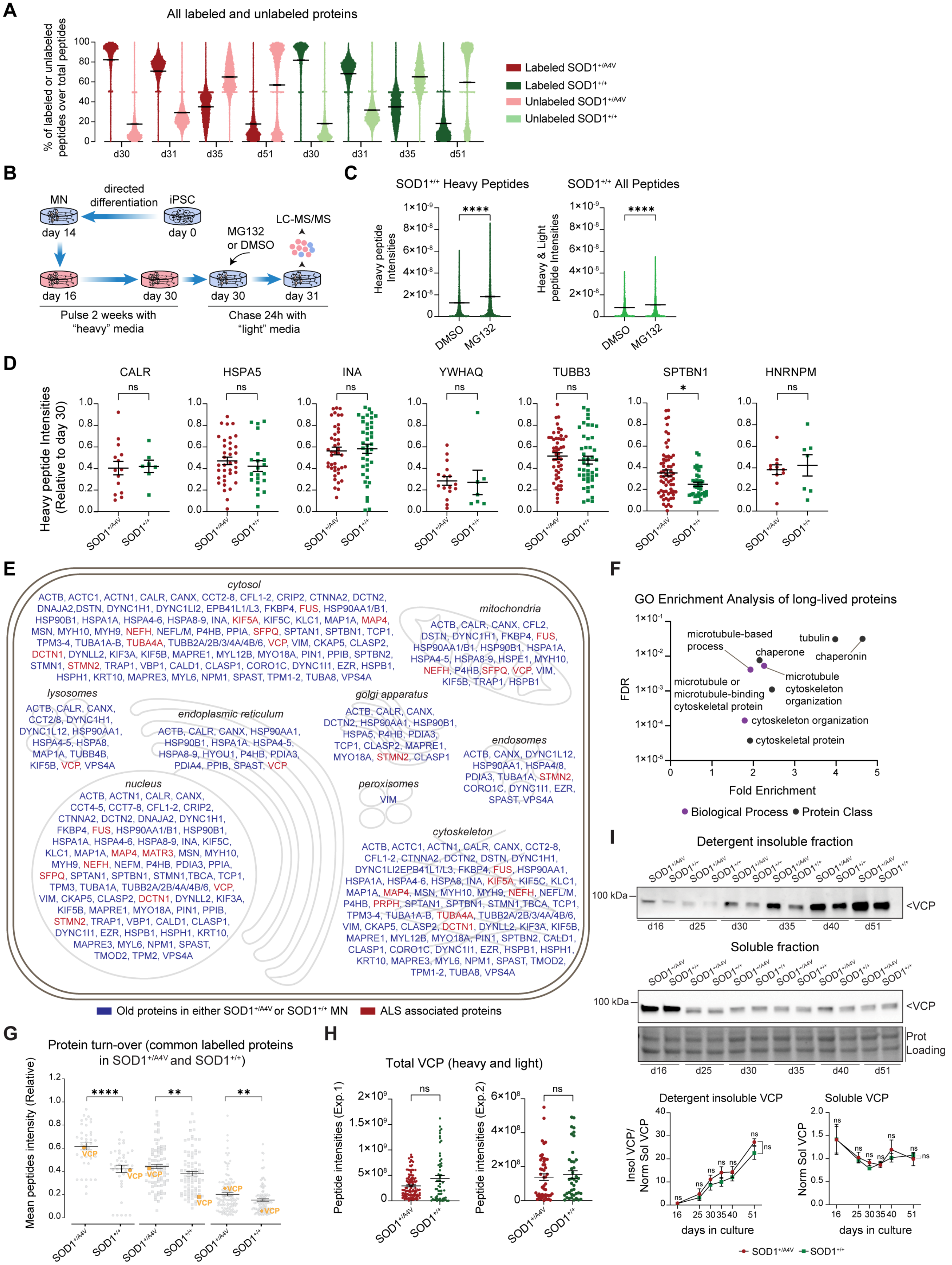
A
All labeled and unlabeled proteins
% of labeled or unlabeled peptides over total peptides
d30 d31 d35 d51 d30 d31 d35 d51
Labeled SOD1+/A4V
Labeled SOD1+/+
Unlabeled SOD1+/A4V
Unlabeled SOD1+/+
B
MN
directed differentiation
iPSC
day 14
day 0
MG132 or DMSO
LC-MS/MS
day 16
day 30
day 30
day 31
Pulse 2 weeks with "heavy" media
Chase 24h with "light" media
C
SOD1+/+ Heavy Peptides
SOD1+/+ All Peptides
Heavy peptide Intensities
Heavy & Light peptide Intensities
DMSO
MG132
D
CALR
HSPA5
INA
YWHAQ
TUBB3
SPTBN1
HNRNPM
Heavy peptide Intensities (Relative to day 30)
SOD1+/A4V
SOD1+/+
E
cytosol
ACTB, ACTC1, ACTN1, CALR, CANX, CCT2-8, CFL1-2, CRIP2, CTNNA2, DCTN2, DNAJA2,DSTN, DYNC1H1, DYNC1LI2, EPB41L1/L3, FKBP4, FUS, HSP90AA1/B1, HSP90B1, HSPA1A, HSPA4-6, HSPA8-9, INA, KIF5A, KIF5C, KLC1, MAP1A, MAP4, MSN, MYH10, MYH9, NEFH, NEFL/M, P4HB, PPIA, SFPQ, SPTAN1, SPTBN1, TCP1, TPM3-4, TUBA1A-B, TUBA4A, TUBB2A/2B/3/4A/4B/6, VCP, VIM, CKAP5, CLASP2, DCTN1, DYNLL2, KIF3A, KIF5B, MAPRE1, MYL12B, MYO18A, PIN1, PPIB, SPTBN2, STMN1, STMN2, TRAP1, VBP1, CALD1, CLASP1, CORO1C, DYNC1I1, EZR, HSPB1, HSPH1, KRT10, MAPRE3, MYL6, NPM1, SPAST, TPM1-2, TUBA8, VPS4A
mitochondria
ACTB, CALR, CANX, CFL2, DSTN, DYNC1H1, FKBP4, FUS, HSP90AA1/B1, HSP90B1, HSPA1A, HSPA4-5, HSPA8-9, HSPE1, MYH10, NEFH, P4HB,SFPQ, VCP, VIM, KIF5B, TRAP1, HSPB1
lysosomes
ACTB, CALR, CANX, CCT2/8, DYNC1H1, DYNC1L12, HSP90AA1, HSPA4-5, HSPA8, MAP1A, TUBB4B, KIF5B, VCP, VPS4A
endoplasmic reticulum
ACTB, CALR, CANX, HSP90AA1, HSP90B1, HSPA1A, HSPA4-5, HSPA8-9, HYOU1, P4HB, PDIA3, PDIA4, PPIB, SPAST, VCP
golgi apparatus
ACTB, CALR, CANX, DCTN2, HSP90AA1, HSP90B1, HSPA5, P4HB, PDIA3, TCP1, CLASP2, MAPRE1, MYO18A, STMN2, CLASP1
endosomes
ACTB, CANX, DYNC1L12, HSP90AA1, HSPA4/8, PDIA3, TUBA1A, STMN2, CORO1C, DYNC1I1, EZR, SPAST, VPS4A
peroxisomes
VIM
nucleus
ACTB, ACTN1, CALR, CANX, CCT4-5, CCT7-8, CFL1-2, CRIP2, CTNNA2, DCTN2, DNAJA2, DYNC1H1, FKBP4, FUS, HSP90AA1/B1, HSP90B1, HSPA1A, HSPA4-6, HSPA8-9, INA, KIF5C, KLC1, MAP1A, MAP4, MATR3, MSN, MYH10, MYH9, NEFH, NEFM, P4HB, PDIA3, PPIA, SFPQ, SPTAN1, SPTBN1, STMN1,TBCA, TCP1, TPM3, TUBA1A, TUBB2A/2B/4A/4B/6, VCP, VIM, CKAP5, CLASP2, DCTN1, DYNLL2, KIF3A, KIF5B, MAPRE1, MYO18A, PIN1, PPIB, STMN2, TRAP1, VBP1, CALD1, CLASP1, DYNC1I1, EZR, HSPB1, HSPH1, KRT10, MAPRE3, MYL6, NPM1, SPAST, TMOD2, TPM2, VPS4A
cytoskeleton
ACTB, ACTC1, ACTN1, CALR, CANX, CCT2-8, CFL1-2, CTNNA2, DCTN2, DSTN, DYNC1H1, DYNC1LI2EPB41L1/L3, FKBP4, FUS, HSP90AA1, HSPA1A, HSPA4-6, HSPA8, INA, KIF5A, KIF5C, KLC1, MAP1A, MAP4, MSN, MYH10, MYH9, NEFH, NEFL/M, P4HB, PRPH, SPTAN1, SPTBN1, STMN1,TBCA, TCP1, TPM3-4, TUBA1A-B, TUBA4A, TUBB2A/2B/3/4A/4B/6, VIM, CKAP5, CLASP2, DCTN1, DYNLL2, KIF3A, KIF5B, MAPRE1, MYL12B, MYO18A, PIN1, SPTBN2, CALD1, CLASP1, CORO1C, DYNC1I1, EZR, HSPB1, HSPH1, KRT10, MAPRE3, MYL6, NPM1, SPAST, TMOD2, TPM1-2, TUBA8, VPS4A
Old proteins in either SOD1+/A4V or SOD1+/+ MN
ALS associated proteins
F
GO Enrichment Analysis of long-lived proteins
FDR
microtubule-based process
tubulin
chaperone
chaperonin
microtubule or microtubule-binding cytoskeletal protein
microtubule cytoskeleton organization
cytoskeleton organization
cytoskeletal protein
Fold Enrichment
Biological Process
Protein Class
G
Protein turn-over (common labelled proteins in SOD1+/A4V and SOD1+/+)
Mean peptides intensity (Relative)
VCP
SOD1+/A4V
SOD1+/+
d31
d35
d51
H
Total VCP (heavy and light)
Peptide intensities (Exp.1)
Peptide intensities (Exp.2)
SOD1+/A4V
SOD1+/+
I
Detergent insoluble fraction
100 kDa
<VCP
d16 d25 d30 d35 d40 d51
Soluble fraction
Prot Loading
Detergent insoluble VCP
Soluble VCP
Insol VCP/ Norm Sol VCP
Norm Sol VCP
days in culture
SOD1+/A4V
SOD1+/+

Supplementary Figure S2. Assessment of SILAC Approach as a Tool to Study Proteins Degradation Dynamics, Identification of Long-lived Proteins in MN and VCP Solubility. Related to Figure 2.

(A) Related to Figure 2C. All labeled and unlabeled proteins at various time-points in either $SOD1^{+/A4V}$ (red and light red) or $SOD1^{+/+}$ (green and light green) MNs. The proteins are represented as a percentage of labeled over total (labeled and unlabeled) peptides. N=2 independent differentiations.

(B) Schematic of the experimental approach used to validate the accumulation of labeled proteins upon blockage of the 20S proteasomal activity.

(C) The levels of the labeled (left) or the total (labeled + unlabeled) (right) peptides upon treatment with the proteasome inhibitor MG132 (1μM, 24h) or the respective vehicle DMSO in isogenic control MNs. Unpaired t-test (two-tailed), p****<0.0001 for either Heavy (left) or All (right) peptides. The peptides in every condition are pooled from three replicate MS experiments.

(D) Analysis of the additional seven persisting proteins at the level of labeled peptides. Each dot represents a single labeled peptide on day 35, with a value normalized to the respective of the same peptide on day 30. Unpaired t-test (two-tailed), n=2 independent differentiations. CALR p=0.869, HSPA5 p=0.4211, INA p=0.714, YWHAQ p=0.884, TUBB3 p=0.428, SPTBN1 p*=0.010, HNRNPM p=0.678; ns=not significant.

(E) Sub-cellular localization (based on Compartments database) of selected long-lived proteins identified in both genotypes. ALS-associated proteins are highlighted in red.

(F) Over-representation analysis (PANTHER database) of the long-lived proteins detected in both mutSOD1 and isogenic control MNs. FDR<0.05.

(G) The VCP protein turnover is highlighted in orange in a modified version of Figure 2D.

(H) Quantification of the average VCP peptide intensity, corresponding in both labeled and unlabeled peptides, in mutSOD1 and isogenic control MNs. Unpaired t-test (two-tailed), n=2 independent differentiations, Exp.1, p=0.633; Exp.2, p=0.720; ns=not significant.

(I) Representative WB (top) and quantification (bottom) of soluble and detergent insoluble VCP. The detergent insoluble VCP is expressed relative to the normalized soluble VCP. Two-way ANOVA across time and genotype p=0.754 (insoluble) and p=0.761 (soluble), Sidak's multiple comparisons test per time-point; for the insoluble d16 p>0.9999, d25 p=0.9813, d30 p=0.8751, d35 p=0.2807, d40 p=0.8364, d51 p=0.4927; for the soluble d16 p>0.9999, d25 p=0.9992, d30 p=0.9255, d35 p>0.9999, d40 p=0.6544, d51 p=0.9976; n=6 independent differentiations; ns=not significant.

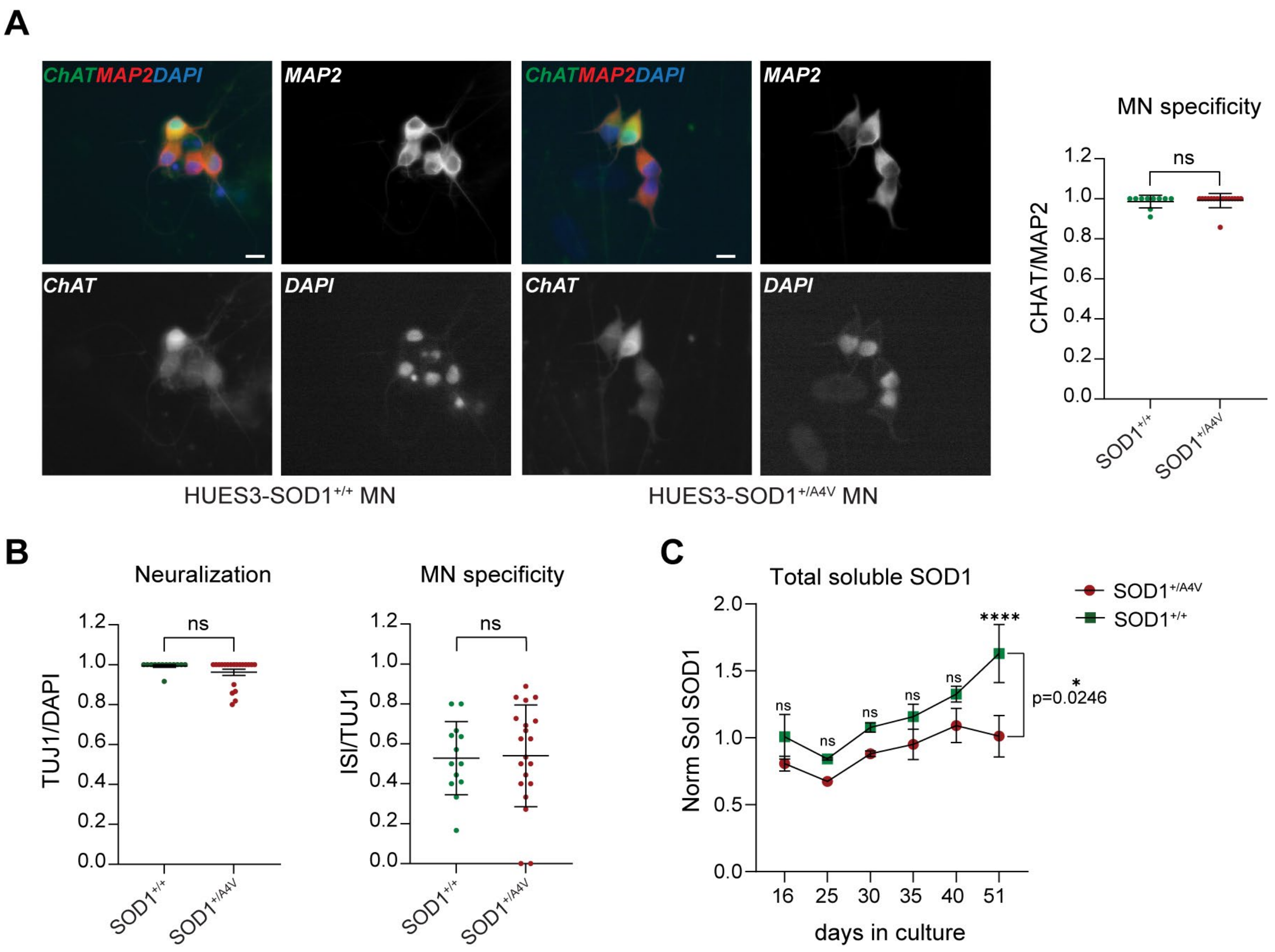

Supplementary Figure S3. HUES3-SOD1$^{+/+}$ and HUES3-SOD1$^{+/A4V}$ derived MN quality control. Related to Figure 3.

(A) Representative images of stem cell-differentiated MNs expressing ChAT and MAP2 on day 25 and assessment of motor neuron specificity (ChAT/MAP2). Unpaired t-test, Mann-Whitney; n=10 for HUES3-SOD1$^{+/+}$ and n=16 for HUES3-SOD1$^{+/A4V}$ independent fields (each field contained more than 20 cells on average); p=0.539. Scale bar = 10μM; ns=not significant.

(B) Assessment of efficiency of neuralization (TUJ1/DAPI) and motor neuron specificity (ISL/TUJ1) on day 25. Unpaired t-test, Mann-Whitney, n=13 for HUES3-SOD1$^{+/+}$ and n=20 for HUES3-SOD1$^{+/A4V}$ independent fields; p=0.196 for TUJ1/DAPI. Unpaired t-test, Welch's correction; n=13 for HUES3-SOD1$^{+/+}$ and n=20 for HUES3-SOD1$^{+/A4V}$ independent fields (each field contained more than 20 cells on average); p=0.878; ns=not significant.

(C) Quantification of SOD1 in the soluble fraction of stem cell derived MNs by WB. Related to Figures 3G-H. Two-way ANOVA across time and genotype p*=0.0246; Sidak's multiple comparisons test per

time-point' d16 p=0.2139, d25 p=0.3800, d30 p=0.2282, d35 p=0.1889, d40 p=0.1079, d51 p****<0.000; n=3 independent differentiations; ns=not significant.

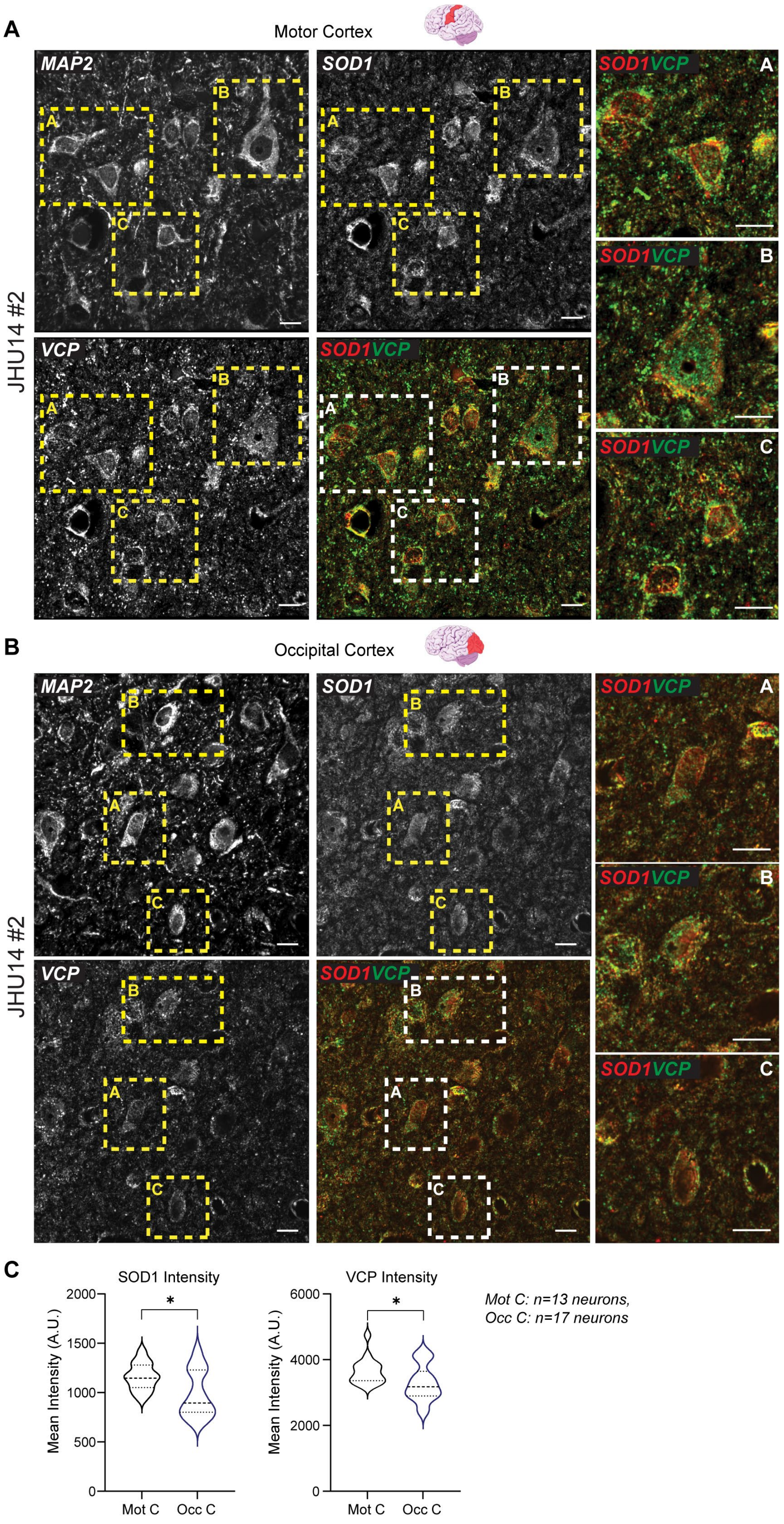
A
Motor Cortex
MAP2
SOD1
VCP
SOD1VCP
A
B
C
JHU14 #2
B
Occipital Cortex
C
SOD1 Intensity
VCP Intensity
Mean Intensity (A.U.)
2000
1500
1000
500
0
6000
4000
2000
*
Mot C
Occ C
Mot C: n=13 neurons,
Occ C: n=17 neurons

Supplementary Figure S4. Postmortem tissue of a second SOD1$^{+/A4V}$ ALS patient shows increased accumulation of VCP. Related to Figure 4.

(A-B) Immunohistochemistry of VCP and SOD1 in postmortem motor **(A)** (affected) and occipital **(B)** (unaffected) cortex tissue from an additional ALS patient (patient #1–JHU14) carrying the A4V mutation in the *SOD1* gene. The pictures on the right column represent the magnified regions within the yellow-dashed squares. Scale bar = 20μM.

(C) Quantification of SOD1 and VCP intensities in MAP2+ neurons within the motor or occipital cortex. Motor cortex n=13 neurons, occipital cortex n=17 neurons. Unpaired t-test (two-tailed); SOD1 p=0.010; VCP p=0.032.

A

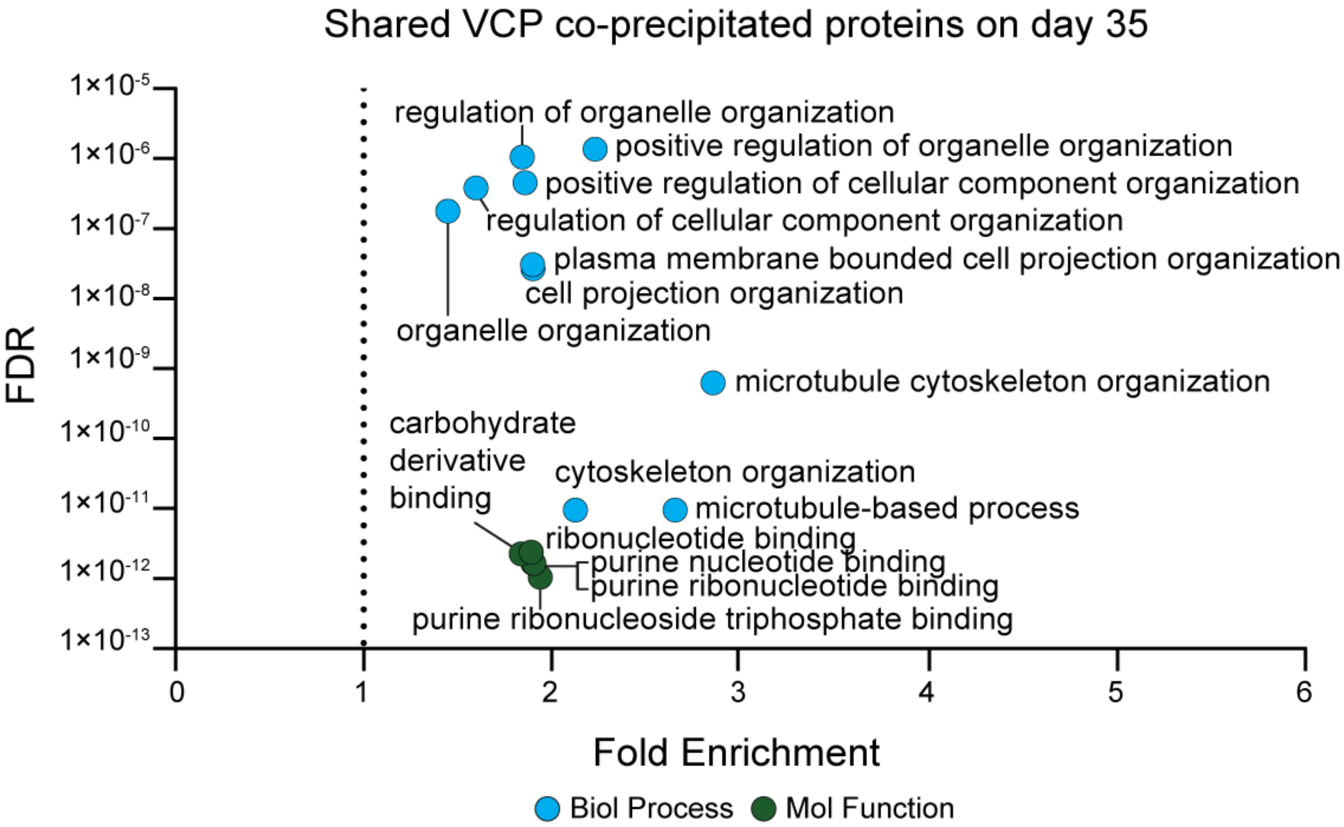

Supplementary Figure S5. Shared VCP co-precipitated proteins. Related to Figure 5.

(A) GO analysis (WebGestalt web tool) of the over-represented groups of the shared VCP-interacting proteins (#399) between SOD1$^{+/A4V}$ and SOD1$^{+/+}$ MNs. In blue are highlighted the groups associated with the Biological Process and in green those with the Molecular Function. FDR<0.05.

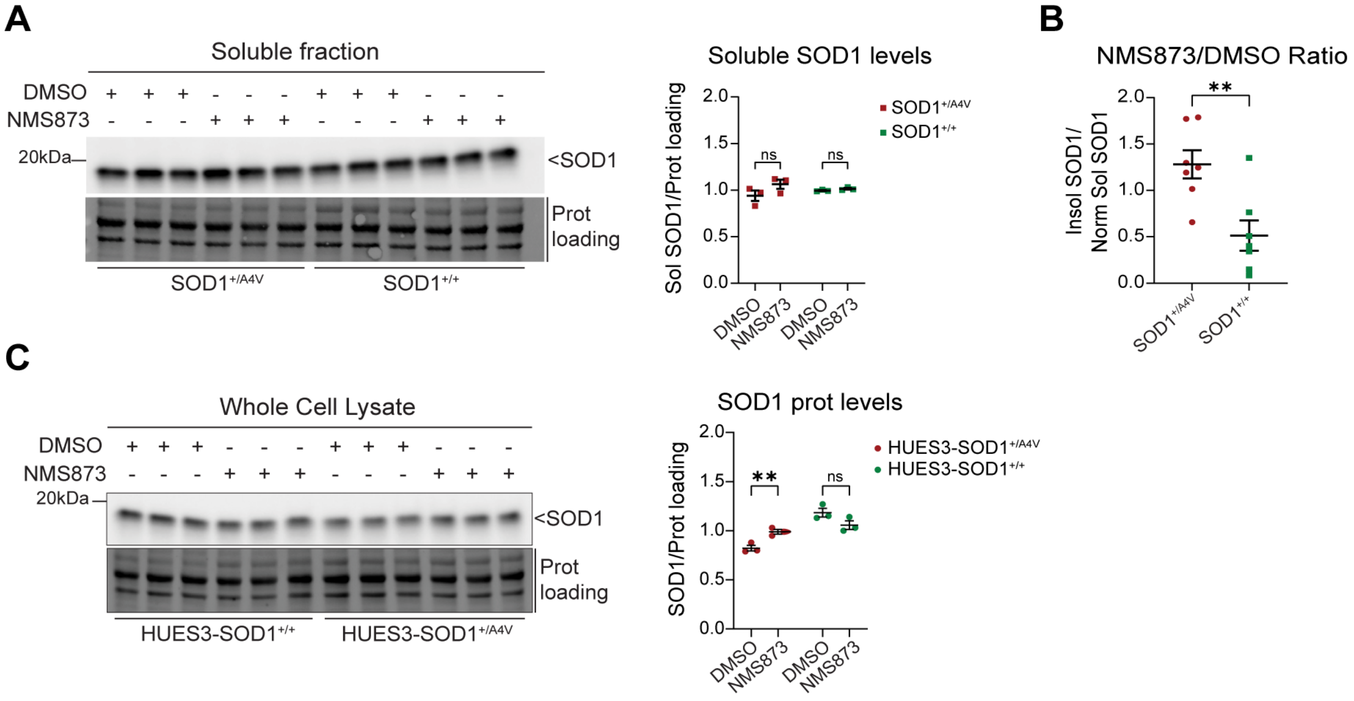

Supplementary Figure S6. VCP impacts the solubility of SOD1. Related to Figure 6.

(A) WB analysis and quantification of soluble SOD1 levels in SOD1$^{+/A4V}$ or SOD1$^{+/+}$ MNs upon treatment with NMS873. Unpaired t-test (two-tailed), SOD1$^{+/A4V}$ MN p=0.1750, SOD1$^{+/+}$ MN p=0.1451; ns=not significant; n=3 biological replicates.

(B) The levels of detergent insoluble SOD1 upon NMS873 treatment, normalized to the respective levels upon DMSO treatment. The detergent insoluble SOD1 is expressed relative to the normalized soluble SOD1. Unpaired t-test (two-tailed), n=7 independent differentiations; p**=0.0049.

(C) WB analysis and quantification of SOD1 protein levels in HUES3 or HUES3-SOD1$^{+/A4V}$ MNs treated with either NMS873 or DMSO. Two-way ANOVA (treatment x genotype) p*=0.0235; Sidak's multiple comparisons test per treatment; HUES3-SOD1$^{+/A4V}$ MN p*=0.0212, HUES3 MN p=0.0707; ns=not significant; n=3 biological replicates.

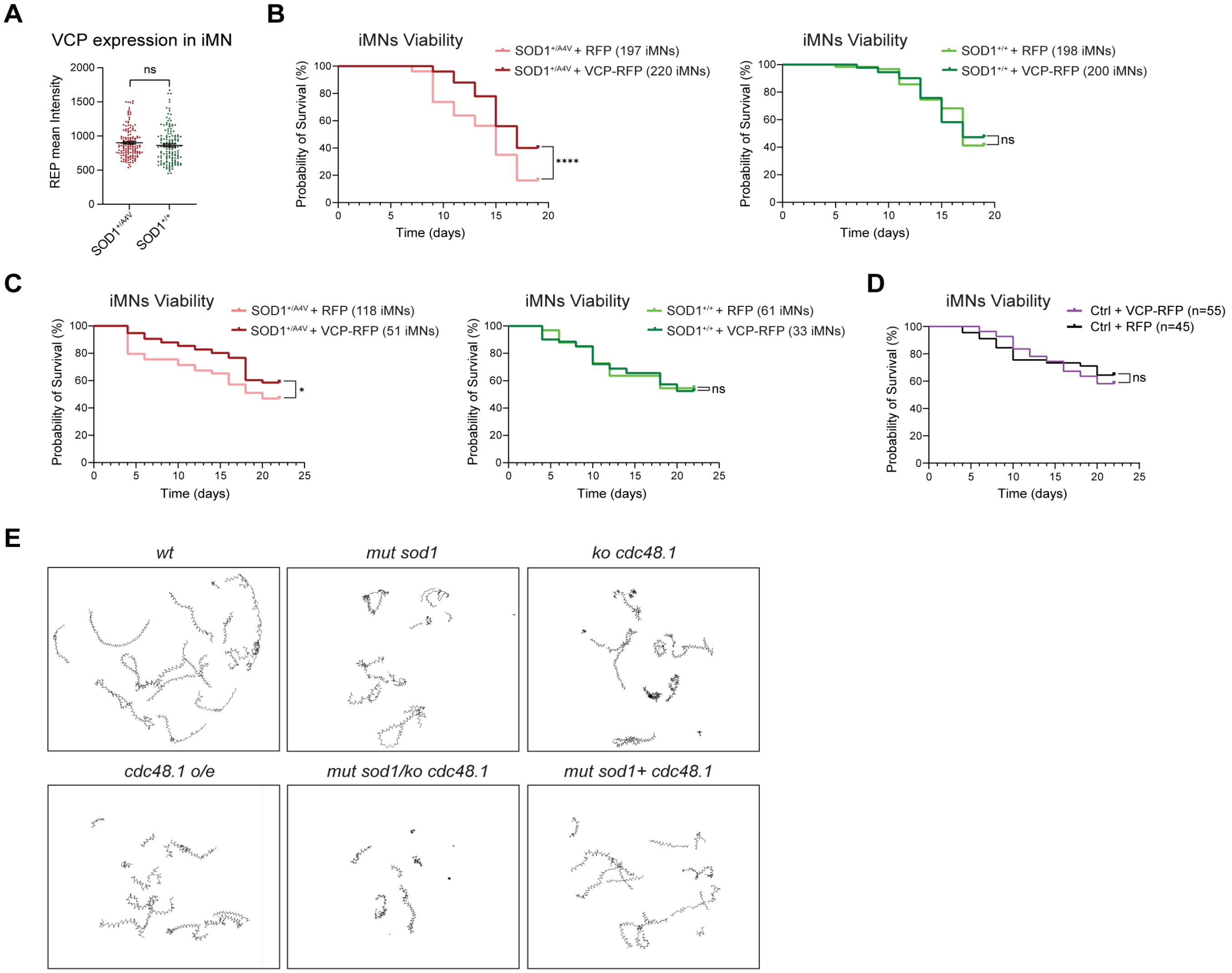

Supplementary Figure S7. VCP Ameliorates Mutant SOD1 Toxicity in iPSC-MNs *in Vitro* and *C. Elegans* Models *in Vivo.* Related to Figure 7.

(A) Quantification of exogenous VCP expression level based on the RFP intensity in mutSOD1 and isogenic control iMNs; unpaired t-test, p=0.071; n=1 differentiation, n=150 infected neurons per genotype; ns=not significant.

(B) The impact of VCP on the viability of mutSOD1 (red, left) or isogenic control (green, right) iMNs. Both plots derive from Figure 7B.

(C) The impact of VCP on the viability of mutSOD1 (red, left) or isogenic control (green, right) iMNs, as quantified in a second independent assay. Gehan-Breslow-Wilcoxon test, for SOD1$^{+/A4V}$ iMNs p*=0.0369, for SOD1$^{+/+}$ iMNs p=0.9280; ns=not significant.

(D) Probability of survival of iMNs generated from a healthy control individual upon VCP-RFP or RFP expression across 3 weeks in culture; n=1 differentiation, for CTRL iMN *LV-VCP-T2A-RFP* n=55, *LV-RFP n=45,* Gehan-Breslow-Wilcoxon test, p=0.831; ns=not significant.
(E) Representative mobility tracks of the various *C. elegans* strains quantified in Figure 7D using Fiji software.

Supplemental Excel Table Titles and Legends

Table S1. Long-lived proteins.
(A) All the proteins that remain labeled up to day 51 in either mutant or isogenic control MNs are listed. The average heavy intensity of each protein expresses the average value of the heavy peptide intensities (corresponding to the same protein) in n=2.
(B) Over-representation analysis of long-lived proteins in both genotypes, showing the enriched groups (FDR <0.05) in the categories of Biological Process and Protein Class (Panther database).

Table S2. SOD1$^{+/A4V}$ MN persisting proteins.
(A-C) Comparison of the common proteins (detected in both mutant and isogenic control MNs) on day 31 **(A)**, day 35 **(B)** and day 51 **(C)**. Note that the average heavy intensity of each protein within each time-point is expressed relative to its respective value on day 30 where the "chase" starts. N=2.
(D) The proteins with higher heavy intensity relative values in patient MNs were pooled from all the three time-points of interest.
(E) Over-representation analysis of persisting proteins in patient MNs, showing the enriched groups (FDR <0.05) in various GO categories (Panther database).

Table S3. VCP-interacting proteins.
(A-C) List of VCP-interacting proteins detected in both genotypes (shared) **(A)**, enriched in patient MNs **(B)** or enriched in isogenic control MNs **(C)**. The spec count value of each precipitated protein is normalized to the respective value of the VCP (bait).

Table S4. Samples analyzed by LC-MS/MS and deposited at MassIVE database.
List of the experimental samples that were subjected to LC-MS/MS.
